# Comprehensive Review of Deep Learning Approaches for Single-Image Super-Resolution

**DOI:** 10.3390/s25185768

**Published:** 2025-09-16

**Authors:** Zirun Liu, Shijie Jiang, Shuhan Feng, Qirui Song, Ji Zhang

**Affiliations:** 1 Longmen Laboratory, Luoyang 471000, China; 23s134148@stu.hit.edu.cn; 2 School of Architecture and Design, Harbin Institute of Technology, Harbin 150001, China; 3 School of Information Engineering, Henan University of Science and Technology, Luoyang 471000, China; 9906685@haust.edu.cn (S.J.); 241404180104@stu.haust.edu.cn (S.F.); 241404180117@stu.haust.edu.cn (Q.S.)

**Keywords:** single-image super-resolution, deep learning, image quality assessment

## Abstract

Single-image super-resolution (SISR) is a core challenge in the field of image processing, aiming to overcome the physical limitations of imaging systems and improve their resolution. This article systematically introduces the SISR method based on deep learning, proposes a method-oriented classification framework, and explores it from three aspects: theoretical basis, technological evolution, and domain-specific applications. Firstly, the basic concepts, development trajectory, and practical value of SISR are introduced. Secondly, in-depth research is conducted on key technical components, including benchmark dataset construction, a multi-scale upsampling strategy, objective function optimization, and quality assessment indicators. Thirdly, some classic SISR model reconstruction results are listed and compared. Finally, the limitations of SISR research are pointed out, and some prospective research directions are proposed. This article provides a systematic knowledge framework for researchers and offers important reference value for the future development of SISR.

## 1. Introduction

**Figure 1:**
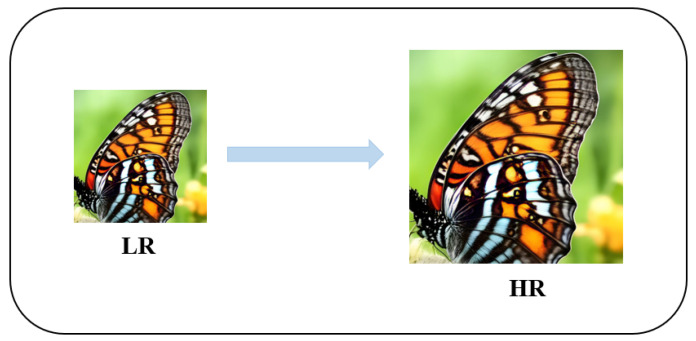
SISR aims to reconstruct a HR image from its degraded LR one.

Image super-resolution (SR), especially single-image super-resolution reconstruction (SISR), is an important research direction in the field of image processing. As shown in Figure 1, the core goal of SISR is to restore high-fidelity high-resolution (HR) images from degraded low-resolution (LR) inputs through advanced algorithm models. This technology has demonstrated significant value in multiple cutting-edge fields, such as image enhancement in security monitoring, medical imaging reconstruction, real-time video resolution enhancement, and precise segmentation tasks in computer vision.

Early research work focused on traditional interpolation-based methods, including bicubic interpolation and Lanczos interpolation [1]. However, due to certain limitations in the use of SISR, the phenomenon of different HR images corresponding to the same LR image always occurs. Subsequently, numerical methods such as edge-based methods [2] and image statistical models [3] have been proposed to address this deficiency. Meanwhile, with the continuous research on SISR, learning-based methods such as neighbor embedding [4] and sparse coding [5] have emerged to learn the mapping between LR and HR image blocks and improve the reconstruction quality.

With the emergence and development of deep learning (DL) [6], compared with other models, it has shown unique advantages and is in a dominant position in many artificial intelligence fields, such as computer vision [7], speech recognition [8], and natural language processing [9]. Based on this, SISR is also constantly developing. The end-to-end framework that can be implemented through DL integrates and unifies the three key steps in SISR tasks—feature extraction, nonlinear mapping, and image reconstruction. In this framework, the three main processes of SISR tasks can be easily implemented, while also helping to improve the performance of SISR and effectively reducing the cost of manual design and calculation. In addition, due to the efficient hardware and complex algorithm support of DL, in order to further promote the development of SISR, more and more researchers are focusing on SISR based on deep learning.

With the development of SISR, more and more SISR methods have been proposed. This article intends to provide a comprehensive review of SISR methods. At present, most existing comments are classified according to supervised methods, while there are relatively few classifications guided by other directions. This article adopts a goal-oriented classification method to classify deep learning-based SISR methods into three categories: “simulated SISR”, “real-world SISR”, and “domain-specific applications”. This classification has wide applicability. It covers simulated scenarios, real-life scenarios, and applications in specific fields. This classification structure based on different objectives is clearer, allowing for a better understanding of the correlations between similar SISR models, making it more interpretable. For example, in the “Efficient Network Design Methods” section, ‘Transformer-based methods” and “Mamba-based methods” are listed, which not only clearly demonstrates their technical relevance but also highlights the core objectives of these two methods in SISR. These two methods not only enable technological innovation in SISR but also strike a balance between efficiency and accuracy. In the “Specific Field Applications” classification section, SISR methods for specific fields, such as medical imaging and remote sensing imaging, are classified. This classification helps researchers to understand that methods in specific fields need to be combined with scene features, thereby enhancing the practical application of SISR models.

**Figure 2:**
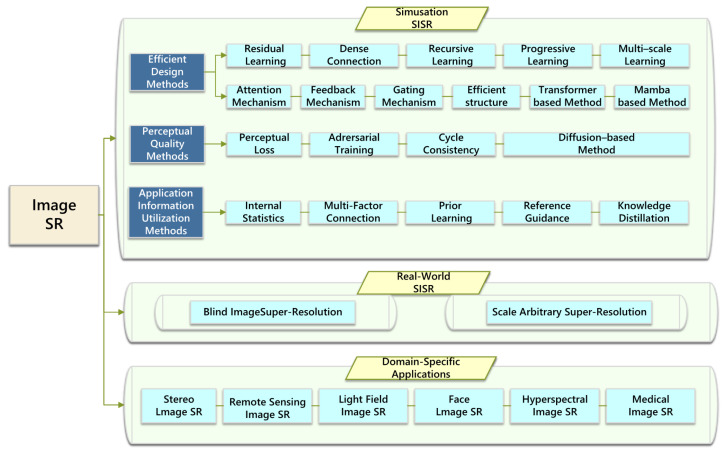
The basic SISR method classification in this review is mainly based on three types: simulated SISR, real-world SISR, and applications in specific fields.

In this article, firstly, the key content of SISR is systematically integrated, and relevant work is analyzed in depth. Secondly, the SISR method is analyzed from the perspectives of simulating SISR, real-world SISR, and specific field applications, as shown in Figure 2. Finally, we discuss the key challenges in SR reconstruction and propose potential research directions by integrating the current development status. The main contributions of this review can be summarized in terms of four aspects:

(1) This article provides an overview of DL-based SISR methods and introduces various SR methods, elaborating in detail on the current status of SR method development;
(2) This article analyzes emerging SISR methods and lists various applications in specific fields, such as interdisciplinary SISR methods related to medical imaging and remote sensing;
(3) We compare the reconstruction results of some SISR models and compare their performance to provide an analysis that is simple and intuitive;
(4) We analyze the technical bottlenecks in the SISR field and explore the future development directions of SR technology by combining strategic frameworks such as cross-modal fusion.

## 2. Problem Statement and Related Works

### 2.1. Problem Statement

Image SR technology aims to improve the resolution of imaging systems, and it can be divided into two types: SISR and multi-image super-resolution (MISR). The core difference between these two types of image SR lies in the number of input images. Comparing the two, it can be found that SISR faces greater technical challenges because it relies entirely on the intrinsic information of a single frame for feature reconstruction, while MISR can utilize complementary information from multiple frames to assist in recovery.

Let the LR image be $I_{x}\in R^{h\times w}$ and its corresponding ground-truth HR image be represented as $I_{y}\in R^{H\times W}$, where the spatial resolution satisfies H>h and W>w. In a typical SISR framework, LR image $I_{x}$ is represented by the degradation process $I_{x}=D\left(I_{y};\theta_{D}\right)$, where $D:R^{H\times W}\to R^{h\times w}$ defines the degradation function parameterized by $\theta_{D}$. The most widely used formula in existing degradation models is

(1) $$D\left(I_{y};\theta_{D}\right)=\left(I_{y}⊗k\right){↓}_{s}+n$$

Among them, LR images are usually generated from high-resolution HR images through blurring, downsampling, and noise interference. $I_{y}⊗k$ corresponds to the convolution operation between the blur filter *k* and the HR image input $I_{y}$. ${↓}_{s}$ represents the subsequent downsampling process with a scaling factor *s*, and *n* usually refers to additive white Gaussian noise (AWGN), characterized by the standard deviation σ.

For SISR, the main objective involves reconstructing SR image $I_{S}R$ from its low-resolution counterpart $I_{x}$. This reconstruction problem is mathematically represented as $I_{S}=F\left(I_{x};\theta_{F}\right)$, which defines the end-to-end SR model, and $\theta_{F}$ represents the trainable parameter set that controls the reconstruction process. In recent research, SISR has been redefined as an end-to-end learning task and relies on the unique advantages of convolutional neural networks (CNNs), further driving the development of DL-based models. Therefore, SISR can be mathematically expressed as the following optimization problem:

(2) $${\hat{\theta }}_{F}=\underset{\theta_{F}}{\arg\min}\;L\left(I_{SR},I_{y}\right)+\lambda \Phi \left(\theta \right),$$

where L represents the difference between the SR output $I_{SR}$ and the ground-truth high-resolution image $I_{y}$, also known as the loss function. Φ(θ) imposes constraints on the model parameters θ, and λ serves as a regularization hyperparameter that balances the trade-off between reconstruction fidelity and model complexity.

### 2.2. Benchmark Datasets

**Figure 3:**
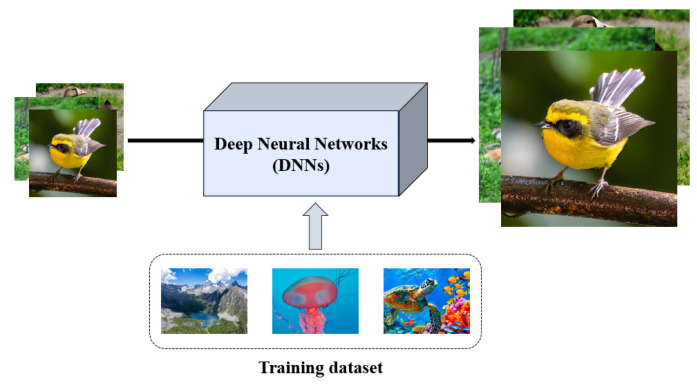
The training procedure for data-driven deep neural networks.

In deep learning-based SISR models, data are crucial in achieving excellent reconstruction performance (as shown in Figure 3). In recent years, both academia and industry have released numerous datasets applicable to SISR.

#### 2.2.1. Degradation Mode

In research, SR datasets still face significant challenges in terms of scale, authenticity, and pairwise matching. In order to integrate data, the existing method is to degrade the dataset to obtain degraded images and then pair them with the original dataset to obtain paired data. In order to improve the authenticity of the dataset and efficiently train models, three commonly used degradation modes have been proposed, namely BI, BD, and DN. With the deepening of research, new degradation modes continue to emerge. For example, some studies consider more complex imaging processes and incorporate factors such as lens distortion and illumination changes into the degradation models.

#### 2.2.2. Training and Test Datasets

In recent years, there have been many datasets used for SISR tasks, such as BSDS300 [10], DIV2K [11], and Flickr2K [12]. At the same time, there are also some test datasets, such as Set5 [13], Set14 [14], Urban100 [15], and Manga109 [16], which can be used to test the performance of the model. These datasets that can be used for SISR are shown in Table 1. Regarding these datasets, DIV2K [11] is widely used for training models, with a total of 1000 images, including 800 training images, 100 validation images, and 100 test images. The Flickr2K [12] training dataset has a large scale, mainly consisting of 2650 images. Urban100 [15] belongs to the group of testing datasets and mainly consists of real-world images, with a total of 100 images.

Recently, new datasets have been proposed. In [17], the authors focus on SISR tasks in specific fields, such as medical image SR. Their dataset consists of three different low-resolution and high-resolution image pairs, promoting the application and success of scanning confocal microscopy in medicine. This provides strong support for the training of more professional and accurate SISR models.

### 2.3. Upsampling Methods

The framework of the SISR method consists of two parts: nonlinear mapping learning and an upsampling module. According to the different stages of upsampling, the framework of the method is divided into four components: pre-upsampling SR, post-upsampling SR, progressive upsampling SR, and iterative up-and-down sampling SR.

The pre-upsampling SR passes through the upsampling module and the nonlinear mapping learning module successively. It avoids nonlinear mapping learning in the low-dimensional space, but it will generate large amounts of blurring and noise, and it has a high cost. Post-upsampling SR is the opposite to pre-upsampling SR. It first performs nonlinear mapping learning in the low-dimensional space and then passes through the upsampling module, greatly improving the efficiency. Progressive upsampling SR combines multiple modules and gradually enlarges the image. It is powerful, but the design is complex and the training is difficult. Iterative up-and-down sampling SR repeatedly performs LR-HR mapping learning. It is complex in design and not very practical. This section mainly explains common upsampling methods.

#### 2.3.1. Based on Interpolation Upsampling Methods

Interpolation is the most commonly used upsampling method, and there are three main methods: nearest-neighbor interpolation, bilinear interpolation, and bicubic interpolation.

Nearest-neighbor interpolation is the simplest interpolation algorithm. It selects the pixel value located at the nearest point to the insertion pixel as the pixel value of the insertion pixel. It simply enlarges the image, and there is no gradual change process between blocks. Its advantage is that the algorithm is simple and fast, but it will generate block effects and obvious visible jagged edges.

**Figure 4:**
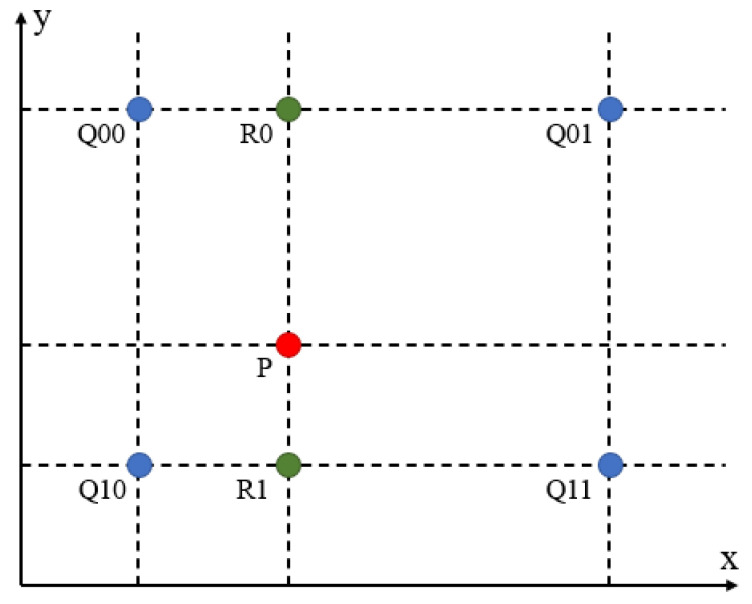
Bilinear interpolation.

Bilinear interpolation involves performing linear interpolation in both the horizontal and vertical directions. Bilinear interpolation needs to find the four nearest pixels around the pixel point to be calculated and calculate the pixel value of the pixel point based on their pixel values and the distance between them. As shown in the Figure 4, in the horizontal direction, two linear interpolations are performed to calculate the pixel values of R0 and R1; then, in the vertical direction, based on the pixel values of R0 and R1, linear interpolation is performed to calculate the pixel value of point P. The formula is as follows:

(3) $$\begin{array}{c} f\left(P\right)=\frac{\left(x_{2}-x\right)\left(y_{2}-y\right)}{\left(x_{2}-x_{1}\right)\left(y_{2}-y_{1}\right)}f\left(Q_{11}\right)+\frac{\left(x-x_{1}\right)\left(y_{2}-y\right)}{\left(x_{2}-x_{1}\right)\left(y_{2}-y_{1}\right)}f\left(Q_{21}\right)\\ +\frac{\left(x_{2}-x\right)\left(y-y_{1}\right)}{\left(x_{2}-x_{1}\right)\left(y_{2}-y_{1}\right)}f\left(Q_{12}\right)+\frac{\left(x-x_{1}\right)\left(y-y_{1}\right)}{\left(x_{2}-x_{1}\right)\left(y_{2}-y_{1}\right)}f\left(Q_{22}\right) \end{array}$$

Bicubic interpolation is a more complex algorithm based on polynomial interpolation. It calculates the pixel value based on 16 pixels around the pixel point to be calculated. It further considers the influence of the change rate of gray values of adjacent pixels. Compared with the previous two interpolation methods, bicubic interpolation can generate smoother and more detail-retained images, but it is relatively complex and slow.

#### 2.3.2. Based on Learning Upsampling Methods

Unlike interpolation methods, which are fixed, frameworks based on learning methods are trained autonomously and can be continuously adjusted to find suitable convolution kernels.

**Figure 5:**
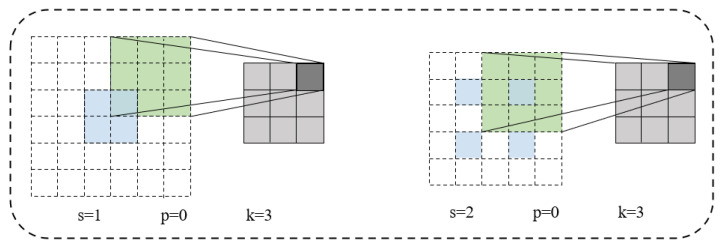
Transposed convolutional layer.

Transposed convolution layers, based on the size of the output pixel and the original pixel, insert zeros in the original pixels around and between (pixels between can also not be inserted) by setting different strides, paddings, and kernels. The relationship between the set stride (s), padding (p), and kernel (k) and the insertion of zeros is as follows: fill s-1 rows and columns with zeros between the elements of the feature map, and fill k-p-1 rows and columns with zeros around the feature map, as shown in Figure 5. Then, through convolution, HR is obtained. However, the transposed convolution layer has a “non-uniform overlap effect”, which will cause the generated image to have a checkerboard texture.

**Figure 6:**
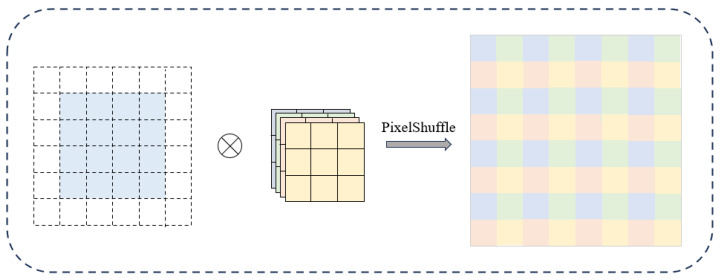
Sub-pixel convolutional layer.

Sub-pixel convolutional layers, as shown in Figure 6, use multiple convolution kernels to perform convolution on the input feature map. First, through convolution, the input feature map of size H × W × C is changed to a feature map of size H × W × ($C*r^{2}$). Through a PixelShuffle operation, the $r^{2}$ channels of each pixel point are rearranged to form an r × r-sized region. Then, each pixel point formed region is combined to output a feature map of Hr × Wr × C. The sub-pixel convolution layer has high computational efficiency and resolves the drawback of transposed convolution generating checkerboard textures, but there will be unnatural textures between small regions, especially for images with severely lost information, and the recovery effect of sub-pixel convolution layers is limited.

### 2.4. Optimization Objective

Evaluation and parameter updates are critical steps for all DL-based models. In this section, we will detail the necessary procedures during model training, including the design of learning strategies and loss functions.

#### 2.4.1. Learning Strategy

In SISR tasks, the choice of learning strategy is crucial for model performance. Based on whether paired low-resolution (LR) and high-resolution (HR) images are used for training, SISR models are mainly divided into two categories: supervised learning methods and unsupervised learning methods.

Supervised Learning. In supervised learning, models are trained using paired LR-HR images. In simulated SISR tasks, LR images are obtained by downsampling HR images, while, in real-world SISR tasks, LR and HR image pairs are acquired by adjusting the camera zoom. The core goal of supervised learning is to minimize the error between the reconstructed image and the ground-truth HR image. The optimization objective of supervised learning can be expressed as

(4) $$\begin{array}{c} \theta_{f}=\underset{\theta }{\mathrm{argmin}}L\left(I_{SR},I_{HR}\right) \end{array}$$

where L is the loss function, and the reasonable selection of the loss function is crucial for model performance.

Unsupervised Learning. Due to the difficulty of obtaining paired LR-HR images, unsupervised learning methods have gradually gained attention. These methods do not require paired LR-HR images but instead use unpaired LR images or self-generated HR images for training. Common unsupervised learning methods include generative adversarial network (GAN)-based methods and self-supervised learning methods. Relevant examples include zero-shot SR (ZSSR) [18], which constructs training data using the test image and its downscaled versions, optimizing the model through data augmentation and loss functions, and CycleGAN [19], which learns bidirectional mappings from LR to HR and HR to LR to achieve unsupervised image SR.

The optimization objectives of unsupervised learning typically involve adversarial loss, cycle consistency loss, and so on, to generate visually realistic HR images. The advantage of unsupervised learning lies in its ability to better adapt to real-world scenarios, although its performance often depends on the accuracy of the degradation model.

#### 2.4.2. Loss Function

The loss function plays a guiding role in optimizing models in SISR tasks. A single loss function often fails to comprehensively reflect the quality of image reconstruction, so researchers usually combine multiple loss functions to guide model optimization, thereby better reflecting the image restoration effect. Below are several commonly used loss functions.

Pixel Loss. Pixel loss is the most basic loss function, used to measure the pixel-level differences between the reconstructed image and the HR image. Common pixel losses include the L1 loss, mean squared error (MSE) loss, and Charbonnier loss. Among them, the L1 loss calculates the absolute difference in pixel values but is less sensitive to outliers, while the MSE loss calculates the mean squared difference in pixel values and is sensitive to outliers. The Charbonnier loss is a differentiable variant of the L1 loss with better numerical stability, often used for noise handling. The pixel loss can be expressed as follows:

(5) $$\begin{array}{c} L_{\mathrm{pixel}}=\frac{1}{HWC}\sum_{i=1}^{H}\sum_{j=1}^{W}\sum_{k=1}^{C}\left|I_{\mathrm{SR}}\left(i,j,k\right)-I_{\mathrm{HR}}\left(i,j,k\right)\right| \end{array}$$

where *H*, *W*, and *C* are the height, width, and the number of channels of the image. The pixel loss is still widely adopted. However, this type of loss function has certain limitations. The reconstructed images lose certain high-frequency details, such as subtle changes in texture and clear contours of edges, resulting in a lack of layered perception.

Adversarial Loss. Adversarial loss is introduced through generative adversarial networks (GANs) [20] to generate more realistic HR images. A GAN consists of a generator and a discriminator, where the generator is responsible for generating fake samples, and the discriminator evaluates the authenticity of the samples. For example, SRGAN [21] proposes a discriminator loss function based on cross-entropy. The adversarial Loss can be expressed as follows:

(6) $$\begin{array}{c} L_{adv}=E_{I_{HR}}\left[\log D\left(I_{HR}\right)\right]+E_{I_{LR}}\left[\log\left(1-D\left(G\left(I_{LR}\right)\right)\right)\right] \end{array}$$

where E represents the mathematical expectation, G stands for the generator, and D denotes the discriminator.

Fourier Space Loss. The Fourier space loss transforms the image into the frequency domain using the fast Fourier transform (FFT) [22] and calculates the differences in the magnitude and phase of frequency components. This loss function can better handle high-frequency information in images, while enhancing the details of reconstructed images. The Fourier space loss can be expressed as follows:

(7) $$\begin{array}{c} L_{\mathrm{fourier}}=\frac{1}{HW}\sum_{u=1}^{H}\sum_{v=1}^{W}\left|Y_{u,v}^{SR}-Y_{u,v}^{HR}\right|+∠\left(Y_{u,v}^{SR},Y_{u,v}^{HL}\right) \end{array}$$

where ($Y_{u,v}$) represents the frequency spectrum of the image.

Prior Loss. Prior loss guides model optimization by incorporating prior knowledge of the image, such as sparsity, gradients, and edges. Common prior losses include the gradient prior loss and edge prior loss. The prior loss can be expressed as follows:

(8) $$\begin{array}{c} L_{\mathrm{prior}}=\sum_{i=1}^{N}\lambda_{i}L_{{\mathrm{prior}}_{i}}\left(I_{SR},I_{HR}\right) \end{array}$$

where *N* represents the number of prior losses, and $\lambda_{i}$ represents the weight coefficient of the *i*th prior loss.

Mixed Loss. To balance the quality, detail, and visual perception of the generated images, researchers often combine multiple loss functions. Below, several commonly used combinations of loss functions are listed.

L1+ perceptual loss combines the pixel-level loss and content loss to generate images with rich details; L1+TV loss combines the pixel-level loss and total variation (TV) loss to reduce blocky artifacts in images; content loss+adaptive loss combines the content loss and adaptive weight loss to generate images with better visual consistency.

Combining the commonly used loss functions mentioned above, such as the pixel loss, content loss, adversarial loss, Fourier space loss, and prior loss, the mixed loss function can be expressed as follows:

(9) $$\begin{array}{c} L_{\mathrm{mixed}}=\lambda_{1}L_{\mathrm{pixel}}+\lambda_{2}L_{\mathrm{content}}+\lambda_{3}L_{\mathrm{adv}}+\lambda_{4}L_{\mathrm{prior}} \end{array}$$

where $\lambda_{1}$, $\lambda_{2}$, $\lambda_{3}$, $\lambda_{4}$ represent different weight coefficients, which are used to balance the importance of various loss terms. Usually, they need to be adjusted to appropriate values through experiments.

In recent years, with the continuous development of deep learning techniques, the design of loss functions in SISR tasks has also evolved. The latest advancements in SISR technology have opened up new paths for its future development. Below, we list some of the latest research advancements.

Multi-Scale Loss: By calculating loss functions at different scales, one can better capture global and local information from the image, improving the quality of reconstructed images. Adaptive Loss Weighting: This dynamically adjusts the weights of different loss functions to better balance image quality and detail. Self-Supervised Loss: This utilizes unpaired LR and HR images for training through self-supervised learning, reducing the reliance on paired data. Frequency Domain Loss: Loss functions incorporating frequency-domain information (for example, Fourier transform, wavelet transform) can better handle high-frequency details in images.

These latest loss function design methods have achieved significant performance improvements. In the SISR task, especially when dealing with complex scenes and low-quality input images, the design of learning strategies and loss functions has a significant impact on model performance. In addition, based on the latest loss function design methods, it is also possible to combine multiple loss functions to more effectively balance image quality, detail, and visual perception.

### 2.5. Assessment Methods

#### 2.5.1. Objective Evaluation Methods

Objective evaluation refers to analyzing the objective features of an image through certain algorithms to obtain relevant quality scores. It is relatively low-cost, fast, and straightforward. At present, the two main objective quality evaluation indices are the peak signal-to-noise ratio and structural similarity.

Peak signal-to-noise ratio. The peak signal-to-noise ratio (PSNR) is a commonly used objective criterion for evaluating image quality. It first needs to calculate the mean square error, i.e., calculate the difference between the individual pixels, and then average these differences to obtain the mean square error. Finally, by incorporating this mean square error into the ratio of the peak signal, and through a logarithmic transformation, an intuitive number is obtained, which directly reflects the quality of the image. The mathematical expression for the PSNR is as follows:

(10) $$\begin{array}{c} PSNR=10\log_{10}\left(\frac{MAX^{2}}{MSE}\right)\; \end{array}$$

(11) $$\begin{array}{c} \mathrm{MSE}\left(X,Y\right)=\frac{1}{W\times H}\sum_{i=1}^{W}\sum_{j=1}^{H}\left(X(i,j)-Y(i,j)\right)\; \end{array}$$

where MSE is the mean square error of each pixel in the original image *X* and the reconstructed image *Y*, MAX is the maximum possible pixel value of the image, and *H* and *W* represent the height and width of the image, respectively. PSNR is measured in decibels (dB), and the magnitude of the value reflects the quality of the image. When the PSNR value is above 40 dB, the quality of the image is close to that of the original high-resolution image, and the image quality is very high. When the PSNR value is at 30–40 dB, the quality of the image is good, and the degree of distortion of the image can be observed. When the PSNR value is 20–30 dB, it indicates that the image has a high degree of distortion and poor image quality. When the PSNR value is below 20, the quality of the image is very poor and the image information loss is serious.

The peak signal-to-noise ratio (PSNR) is a widely used and easy-to-understand image quality evaluation metric. By calculating the mean square error between the original image and the reconstructed image, the PSNR can quantify the similarity between them. Higher values indicate better image quality, which makes it possible to visually compare the quality of different image processing methods. The advantage of the PSNR is that the calculation process is simple and clear; it is easy to understand and explain. This intuitive and quantitative evaluation method has led to the widespread acceptance and use of the PSNR in the fields of image processing and communication. However, it is important to note that the PSNR, while simple and easy to understand, does not always fully reflect the perception of image quality by the human visual system, so, in some cases, it is necessary to combine other evaluation indicators to comprehensively evaluate the image quality.

Structural similarity. The structural similarity index (SSIM) [23] is a measure of both original and reconstructed images. Unlike the PSNR, the SSIM is a similarity index designed to more comprehensively consider human perceptions of image quality. Focusing only on luminance information, the contrast and structural information of the image is also considered. The calculation of the SSIM is based on brightness and contrast. The brightness part measures the average brightness of the image, the contrast part considers the range of brightness variation, and the structure part focuses on the texture and structure information of the image. By combining these three aspects, the SSIM can provide more granular and comprehensive information in image quality assessments. The formula for calculating the SSIM based on brightness, contrast, and structure is

(12) $$\begin{array}{cc} l\left(I_{SR},I_{HR}\right) & =\frac{2\mu_{SR}\mu_{HR}+c_{1}}{\mu_{SR}^{2}+\mu_{HR}^{2}+c_{1}} \end{array}$$

(13) $$\begin{array}{cc} c\left(I_{SR},I_{HR}\right) & =\frac{2\sigma_{SR}\sigma_{HR}+c_{2}}{\sigma_{SR}^{2}+\sigma_{HR}^{2}+c_{2}} \end{array}$$

(14) $$\begin{array}{cc} s\left(I_{SR},I_{HR}\right) & =\frac{\sigma_{SRHR}+c_{3}}{\sigma_{SR}\sigma_{HR}+c_{3}} \end{array}$$

(15) $$\begin{array}{cc} \mathrm{SSIM}\left(I_{SR},I_{HR}\right) & ={\left[l\left(I_{SR},I_{HR}\right)\right]}^{\alpha }{\left[c\left(I_{SR},I_{HR}\right)\right]}^{\beta }{\left[s\left(I_{SR},I_{HR}\right)\right]}^{\gamma }\\ \; & =\frac{\left(2\mu_{SR}\mu_{HR}+c_{1}\right)\left(\sigma_{SRHR}+c_{2}\right)}{\left(\mu_{SR}^{2}+\mu_{HR}^{2}+c_{1}\right)\left(\sigma_{SR}^{2}+\sigma_{HR}^{2}+c_{2}\right)} \end{array}$$

where $I_{SR}$ and $I_{HR}$ represent SR and HR images, respectively; $\mu_{SR}$ and $\mu_{HR}$ represent the mean pixels of the SR and HR images; $\sigma_{SR}$ and $\sigma_{HR}$ represent standard deviations; $\sigma_{SRHR}$ represents the covariance of the SR and HR images; $c_{1}$, $c_{2}$, and $c_{3}$ are constants to avoid errors caused by a zero denominator. SSIM can range from zero to one, where a value of 1 indicates that the two images are identical, and a value close to 0 indicates that they are very different. Therefore, the closer the SSIM value is to 1, the more similar the reconstructed image is to the original image, and the higher the image quality. Conversely, the closer the value is to 0, the more significant the differences between the images and the lower the quality.

#### 2.5.2. Subjective Evaluation Methods

In addition to objective evaluation methods, subjective evaluation methods also play a crucial role in image quality evaluation. Although the PSNR and SSIM provide numerical measures, they cannot fully simulate the perception of images by the human visual system. Human subjective perception is influenced by many factors, including cognition, subjective preferences, and esthetics. Therefore, the subjective evaluation method provides an assessment of image quality that more closely reflects real perception by inviting observers to perform actual observations and evaluations. A commonly used subjective evaluation criterion is the mean opinion score (MOS), which is obtained by inviting a group of observers to evaluate an image, and their scores are averaged to obtain a composite score. These scores, which typically range from 1 to 5, represent the observer’s subjective perception of image quality, with higher scores indicating better image quality and lower scores indicating worse quality, as shown in Table 2.

#### 2.5.3. Fine-Grained Error Analyses

Traditional indicators cannot reveal specific behavioral differences in texture, edges, and other aspects during the model image reconstruction process. Researchers call for fine-grained error analysis to further analyze SISR models. In this way, one can devise a classification method for common artifacts such as texture distortion, edge blurring, noise amplification, and false detail generation. Then, experts annotate the main artifacts in the SR output on the annotated benchmark dataset. This method can use confusion matrices to quantify SISR models. For example, the confusion matrix may display the confusion between texture artifacts and edge artifacts, thereby revealing the limitations of the model, considering aspects that were not reflected in the overall score. This can provide precise guidance for improving models and help to solve specific problems, such as texture restoration and edge sharpness.

### 2.6. Ablation Studies for Component Validation

To quantify the contributions of key components in the proposed SISR framework, we conducted ablation experiments using the SRCNN [7] model as an example. This included dataset construction strategies, upsampling modules, loss function design, and evaluation methods.

Specifically, we adjusted the dataset construction strategy, upsampling method, loss function, and evaluation method separately. All experiments were conducted under the same training (T91 dataset) and testing (Set5 dataset, ×2) conditions. The results showed that, when adjusting the dataset construction strategy or changing the degradation kernel, its performance significantly decreased. For the upsampling method, the performance slightly decreases after transpose convolution. For the loss function, using the L1 loss can allow an improvement in performance. Overall, for SRCNN, the dataset construction strategy is the most critical, and the loss function design has a significant impact on the image quality. Upsampling methods are related to efficiency and performance, and diverse evaluation methods can comprehensively reflect changes in model performance. This experimental framework can provide a reference for other models, but different models have different characteristics and they still need to be analyzed to improve model performance.

## 3. Image Super-Resolution

In 2014, the SR Convolutional Neural Network (SRCNN) proposed by Dong et al. [7] was of milestone significance in the field of SISR. As the first SISR model based on a convolutional neural network (CNN), SRCNN revealed the equivalence between the deep CNN architecture and the traditional example-based learning method based on sparse coding in the mathematical framework through theoretical analysis, successfully introducing deep learning into the field of SR research. This groundbreaking work promoted a shift in the research paradigm of SISR. Subsequent studies gradually framed the SR task as an end-to-end deep learning problem, directly establishing a nonlinear mapping relationship between LR images and HR images through deep neural networks. Since then, CNN-based SR methods have entered a stage of rapid development. Innovative techniques such as residual learning and attention mechanisms have been successively introduced, continuously surpassing performance records in objective indicators such as the PSNR and SSIM, as well as in visual perception quality, and continuously driving technological progress in this field.

### 3.1. Simulation SISR

In recent years, SISR reconstruction technology has been developing rapidly, with many innovative algorithm models emerging. It is worth noting that the current mainstream research methods generally adopt the training and validation paradigm based on simulated datasets, which is defined as the “simulated SISR” technical path in the academic community. The core feature of this technical route is to degrade HR images through a preset fixed degradation model (such as bicubic downsampling) and then generate paired LR images as training data. However, the difference between these simulated data and the actual scenario may lead to a performance decline in the model in real-world applications.

It should be particularly pointed out that, despite the above limitations, the simulated SISR method has still had a profound impact on the development of the field. According to the differences in technical routes, existing methods can be systematically divided into three research branches: innovation in efficient network architectures and computational mechanisms, perceptual quality-oriented methods, and multi-modal information fusion technology. These three research directions complement each other and jointly promote theoretical breakthroughs and application expansions in SISR technology.

#### 3.1.1. Efficient Network/Mechanism Design Methods

The techniques that have emerged in the last few years have focused on the efficient and precise design of network architectures and mechanisms, which can be modeled to achieve better performance with fewer parameters. Therefore, the choice of an appropriate research methodology is particularly crucial when studying the dynamical behavior of nonlinear systems.

Residual learning In the development and research of SR convolutional neural networks, it is known that increasing the number of layers in the convolutional neural network can improve the accuracy of model training and thus lead to better results. However, directly stacking too many layers may lead to problems such as “gradient vanishing” and “gradient explosion” [24].

**Figure 7:**
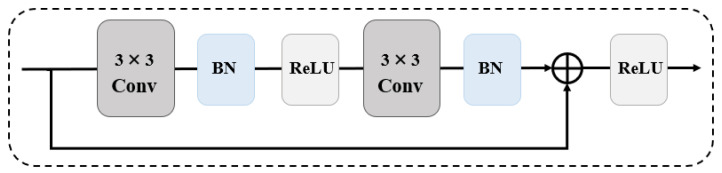
Sketch of residual learning architecture/residual block.

In ResNet [25], He et al. introduced a residual learning framework designed to learn a residual mapping instead of directly approximating the entire underlying function (Figure 7). In SISR, there is a lot of common information between low-resolution (LR) images and high-resolution (HR) images, making it easy to model the relationships between high-resolution and low-resolution images directly. The advantages of residual learning are obvious. It not only allows the number of layers in the neural network to increase but also alleviates problems such as gradient vanishing and network degradation. Kim et al. [26] proposed another deep SR network, VDSR. With the in-depth development of deep learning, to make the network design simpler, residual blocks have gradually become the basic components for building network structures. For example, a convolutional branch usually contains two 3 × 3 convolutional layers, two batch normalization layers, and a ReLU activation function in the middle. However, Lim et al. [27] found through research that, in SISR tasks, batch normalization layers consume a lot of memory but do not significantly improve model performance, so batch normalization layers are generally removed from the network structure. In addition, Lu et al. [28] proposed MARNet, which combines a residual network structure and attention mechanism. It can effectively extract image information and fuse multi-layer features, further improving the reconstruction accuracy.

Global and local residual learning: Global residual learning optimizes the learning process of the entire network by establishing a connection between the input and the final reconstruction layer, improving information transmission, and it can improve the network performance and reduce information loss. However, residual networks usually contain parameterized layers, making the network model more complex and potentially causing the loss of image details. Therefore, local residual learning has been proposed. Local residual learning uses residual connections at the local level rather than from input to output. This multi-path learning method can carry rich image details and enhance the network’s expressive ability. Of course, local residual learning can also be applied in the feature extraction module to enhance the learning ability of the module [29,30]. In modern times, combining global residual learning and local residual learning is highly applicable. Using both can effectively improve the effectiveness of the model.

Residual scaling: Lim et al. [27] proposed an enhanced deep super-resolution network (EDSR) that can improve the performance by expanding the model size, and they also proposed a new multi-scale deep super-resolution system (MDSR) that can reconstruct high-resolution images with different magnification factors in a single model. In GAMSRN [31], this method utilizes global attention multi-scale residual blocks. This method can not only extract features at different scales but also improve the performance without increasing the model complexity.

Dense connection: This connection method is widely used in dense connection convolutional networks and in neural networks. It enables each layer in the dense block to be connected to all previous layers, thereby obtaining the features of the previous layers. The short paths established between these layers can alleviate gradient vanishing and gradient explosion and enhance the information exchange between these layers, further improving the precision of reconstruction.

**Figure 8:**
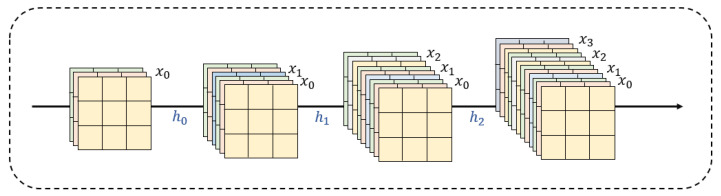
Dense connection module.

In the field of deep learning image reconstruction, Tong et al. [32] proposed SR-DenseNet. SR-DenseNet not only employs hierarchical dense connections to enable the exchange and integration of features at different levels but also introduces block-level dense connections, ensuring that the outputs of each dense block are interconnected through dense connections (Figure 8). This design allows for the effective combination and utilization of low-level and high-level features during the image reconstruction process, thereby enhancing the reconstruction effect. Kuldeep et al. [33] proposed a hybrid dense connection block (MDCB) that combines the advantages of residuals and dense connections, which can achieve the SR of multiple factors, improve the performance, and achieve high factor parameter efficiency. Besides SR-DenseNet and MDCB, the dense connection mechanism has been widely applied in other models, such as MemNet [34], RPMNet [35], and MFNet [36]. By using the dense connection mechanism, the information flow between different depths of the network can be efficiently facilitated and utilized, allowing the model to fully exploit data features and resulting in superior image reconstruction performance.

Recursive learning: Recursive learning methods involve repeatedly applying the same submodules in the same model and using the same data. Among them, each recursive module is composed of many recursive units, which have the same structure and data. However, during the use of recursive learning methods to construct models, if there are many stacked layers, problems such as vanishing or exploding gradients can easily occur. To address this issue, a deep recursive residual network based on residual learning can be constructed to construct recursive blocks. In DSRN [37], it is possible to enhance sequence data processing by introducing a two-state mechanism, a structure that allows the model to maintain two different internal states within the same time step, thus better capturing long-term dependencies and improving the performance. In recent years, an increasing number of models have adopted the idea of residual learning in recursive units, such as MDCB [33], MemNet [34], CARN [38], and SRRFN [39].

Progressive learning: Progressive learning is a strategy that aims to help learners or models to adapt and develop better by gradually increasing the difficulty of a task. It shows excellent performance when dealing with complex sequence prediction and decision-making tasks. It not only shortens the training cycle but also significantly enhances the generalization ability of the model. SISR is a complex and unstable problem, with challenges such as large-scale factorization, undetermined degradation kernels, insufficient data, and noise interference. By adopting an incremental learning strategy, we can split the SISR task into a series of shallow to deep subtasks, starting from simple small-scale problems and gradually increasing their difficulty, which can enhance the performance and generalization ability of the model during continuous challenges and thus effectively simplify the learning process and improve the learning efficiency.

In PRAN [40], it is shown that introducing progressive residual attention for optimization can improve the performance. In the design of ProSR [41], the layers of the pyramid were gradually blended to minimize the impact on the previously trained layers by gradually introducing training pairs for various scales. Wong et al. [42] proposed a progressive adversarial network (PAN) that utilizes U-Net and a neural structure for progressive growth; this can enhance the texture details of U-Net while ensuring the accuracy of image reconstruction. Through progressive learning, the complex problem is decomposed into several smaller subtasks, so that it is possible start from simple problems and gradually accumulate experience, thus accelerating the convergence of the algorithm and showing better results in the reconstruction stage.

**Figure 9:**
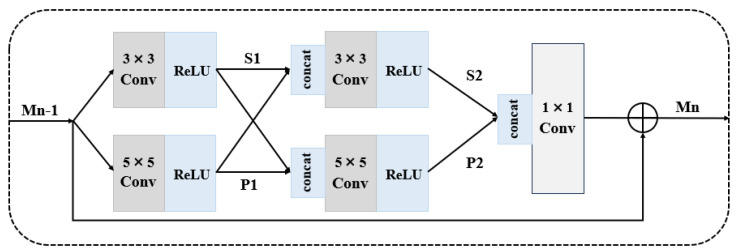
Structure of multi-scale residual block (MSRB) [29].

Multi-scale learning: Enriched image features are a key factor in achieving high-quality image SR reconstruction. In recent years, many research works [40,43,44] have pointed out that images exhibit diverse features in different scenes and conditions. Therefore, fully exploiting and utilizing these diverse features can significantly improve the performance of the model and enhance the effects of image reconstruction. Li et al. [29] proposed a multi-scale residual block (MSRN) for feature extraction; it introduces convolution kernels of different sizes on top of the residual block and adaptively detects image features of different scales while letting these features interact with each other to obtain the most effective image information (Figure 9). After this, Li et al. [45] further optimized the structure and proposed the multi-scale dense cross-network (MDCN); this can maximize the utilization of image feature flows at different scales to improve the performance.

**Figure 10:**
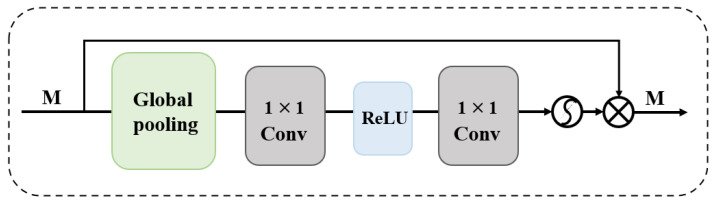
Core operational framework of CAM.

In recent years, multi-scale SISR models have been proposed one after another. For example, Qin et al. [46] proposed a multi-scale feature fusion residual network (MSFFRN) to better exploit the image features in SISR. Chang et al. [47] organically integrated multi-scale learning and dense connectivity techniques to form a novel dual-scale dense network (MSDN). Cao et al. [48] proposed a novel multi-scale residual channel attention network (MSRCAN) and introduced the channel attention mechanism (CAM, Figure 10) in MSRCAN. Yazıcı et al. [49] proposed a multi-scale lightweight hybrid network (GLIMS), which combines the bottleneck structure of Swin Transformer with attention guidance and multi-scale feature aggregation to effectively capture local and global features. Li et al. [50] proposed the remote multi-scale fusion network (LMFN). This network is capable of discovering multi-scale priors in images and utilizing interactive fusion modulation modules to fuse multi-scale features. These examples also show that effectively extracting and utilizing the features of multi-scale images is the key to further enhancing the reconstructed images.

Attention Mechanism: An attention mechanism is a tool that prioritizes the allocation of resources to the most informative input regions, thereby enhancing the learning efficiency. With the increasing popularity of attention mechanisms, similar attention mechanisms have been introduced in the field of SISR. Mei et al. [51] proposed a deep residual network based on squeeze-and-excitation blocks, which can improve the performance and enhance the accuracy of reconstructed images. In order to fully utilize the potential of convolutional neural networks in the field of SR, Yan et al. [52] proposed a multi-scale attention network (MAN) that combines classical multi-scale mechanisms with emerging large kernel attention. The MAN network is comparable to SwinIR in terms of performance and computational efficiency, achieving a different trade-off between optimal performance and computational efficiency.

Traditional attention mechanisms have significantly improved the performance of SISR but often lead to complex network architectures and many parameters, resulting in slow inference speeds and large model sizes. To address this issue, SPAN [53] was proposed, an efficient SISR model that balances the number of parameters, inference speed, and image quality. SPAN employs a novel parameter-free attention mechanism that leverages symmetric activation functions and residual connections to enhance highly contributing information and suppress redundant information. This further demonstrates the effectiveness of attention mechanisms, which help to further improve model performance. Majid et al. [54] proposed a spatial edge residual attention model. This model combines edge and spatial attention to achieve better performance in image reconstruction. Yu et al. [55] proposed an algorithm that utilizes a dual attention module to capture implicit weight information and employs residual connections to fuse global features. This algorithm can allocate computing resources more effectively and accelerate the computing speed.

Feedback Mechanism: Feedback mechanisms enhance the SR reconstruction accuracy by introducing recurrent structures into the model, allowing it to use previous output information to guide subsequent computations. Feedback networks have been applied in SR, enhancing the network’s feature extraction and reconstruction capabilities through feedback mechanisms. In SRFBN [56], the authors introduce feedback connections that allow the network to share and reuse features across different layers. These feedback connections help the network to better learn the global and local features of images, and SRFBN can achieve better performance than existing methods with fewer residual dense blocks.

Gating Mechanism: Gating mechanisms improve the model’s expressive power and computational efficiency by introducing gated units to control the flow of information. Specifically, gating mechanisms can dynamically adjust the transmission of information within the network based on the input features, allowing the model to focus more on important features and suppress unimportant ones, thereby enhancing the reconstructed image. Trishna et al. [57] proposed that a gating module with attention mechanism can be learned to effectively fuse relevant feature images and improve the accuracy of image reconstruction. Yao et al. [58] proposed a lightweight SISR guided by gated feedback and LatticeFormer. This method can improve the feature extraction ability and effectively obtain global features at different levels.

#### 3.1.2. Efficient Structures

Although increasing the depth of the model is a direct method to improve SR performance, its enormous computational cost makes it difficult to deploy deep models in scenarios with limited computing power, such as mobile devices. In order to solve this problem, many efficient SISR solutions have emerged in the academic community in recent years, mainly achieving breakthroughs in two directions: network architecture optimization and knowledge transfer.

In terms of network architecture innovation, Hui et al. [59] were the first to propose the Information Distillation Network (IDN), which effectively compresses the number of parameters by constructing feature distillation channels. Subsequent research further upgraded it to the Information Multi-Distillation Network (IMDN), which significantly improves the feature fusion efficiency by adopting a cascaded distillation block architecture. The RFDN developed by Liu’s team [60] reoptimizes the computational efficiency through lightweight feature distillation connections, while VapSR proposed by Zhou et al. [61] enhances the network’s representational ability through a dynamic routing mechanism. It is worth noting that Liu et al. [62] innovatively designed a collaborative architecture consisting of an efficient residual block (ERB) and a high-frequency attention block (HFAB). The ERB module realizes deep feature learning through reconfigurable skip connections, and the HFAB uses the Laplacian operator for high-frequency feature detection and dynamically enhances detail reconstruction through pixel-level scaling factors.

In the lightweight technology route, the knowledge distillation method shows unique advantages. Lee et al. [63] pioneered the introduction of the teacher–student learning framework into the SISR field. By constructing a lightweight student model to inherit the knowledge distilled from the teacher network, a good balance between computational efficiency and reconstruction quality is achieved. In terms of hardware-adapted architectures, researchers have drawn on the successful experiences of MobileNet and ShuffleNet. Lin et al. [64] replaced standard convolution operations with depth-separable convolutions, which greatly reduces the parameter scale. Sun et al. [65] proposed ShuffleMixer, which has demonstrated the latest breakthrough. This technology, through the collaborative design of large-sized convolution kernels and channel shuffling operations, significantly expands the receptive field while maintaining mobile-end compatibility.

These innovative methods effectively resolve the computational bottlenecks of traditional deep models during mobile-end deployment through architecture improvements and algorithm optimizations, providing important technical support for the practical application of real-time SR technology. Future research can further explore directions such as dynamic network architectures and the joint optimization of hardware awareness to continuously promote the development of lightweight SR technology.

#### 3.1.3. Transformer-Based Methods

The core idea of the Transformer is the "self-attention" mechanism, which can capture the long-range information correlations between token elements. Recently, Chen et al. [66] made innovative improvements to the "self-attention" mechanism and proposed the recursive generalization self-attention. It integrates image features into representative feature maps through a recursive generalization module, achieving the aggregation of global key information. As a result, it can effectively capture global spatial information while maintaining a low computational cost, enhancing the model’s perception of global features.

In recent years, Transformer-based models have emerged in an endless stream. For example, Liu et al. [67] proposed Swin Transformer, which is a highly innovative model. It introduced the sliding window mechanism, including non-overlapping local windows and overlapping cross-windows. By restricting the attention calculation within the windows, the computational amount is greatly reduced. Meanwhile, with the help of the sliding window operation, the attention mechanism can pay attention to global features, improving the performance of the model. Chen et al. proposed the Image Processing Transformer (IPT [68]), which was pretrained on large datasets, and introduced contrastive learning for different image post-processing tasks. Therefore, the pretrained model can be fine-tuned on the required tasks, showing good adaptability.

Meanwhile, Liang et al. proposed SwinIR [69] for image restoration. The Swin Transformer block proposed in this model is used for feature extraction, and DIV2K-Flicker2K was selected as the training set, reducing the dependence on large-scale datasets and being applicable to different scenarios. Zamir et al. [70] proposed Restormer, which reconstructs high-quality images by embedding convolutional neural networks (CNNs) within the Transformer. Subsequently, Chen et al. [71] used overlapping cross-attention modules and combined these with a pretraining strategy to enhance the performance of the Transformer model. Jiang et al. [72] mentioned an image SR reconstruction method based on a lightweight symmetric CNN–Transformer. Through its unique module design, it improves the processing ability for local and global features. Guo et al. [73] proposed the CMTNet model with a dual-branch structure. This model consists of a feature extraction module and an edge enhancement module. The feature extraction module is further divided into a Transformer high-frequency feature extraction branch and a feature extraction branch based on ConvLSTM. The feature fusion of the two branches ensures the order of spectral information, providing new ideas and methods for image SR reconstruction. In addition, Zhang et al. [74] proposed CVIformer in 2025, which is a new architecture that integrates cross-view interaction into Transformer. This architecture can effectively combine input images to restore the details of stereo SR.

#### 3.1.4. Mamba-Based Methods

Mamba is a novel deep learning architecture that enables fast inference and accelerates computation speeds. To some extent, it can replace Transformer and improve efficiency. Recently, He et al. [75] proposed the Mamba Pixel Sequential Interactive Network (MPSI). This network effectively enhances long-distance connections and improves the effectiveness of image reconstruction by using channel Mamba blocks (CMB) and Mamba channel recursive modules (MCRM). Wang et al. [76] proposed a collaborative network consisting of Mamba and a CNN (CNMC), which is mainly used for lightweight image SR. It can reduce model complexity, improve the computational speed, and extract deep features that are beneficial for reconstruction. Romina et al. [77] proposed MambaLiteSR, which mainly utilizes the Vision Mamba architecture and effectively reduces power consumption and performance loss through methods such as knowledge distillation. It is a lightweight image SR model. Jiang et al. [78] proposed Rep Mamba, an architecture based on the RSISR state space model. They designed a cross-scale state propagation mechanism (CSSP) and constructed a lightweight progressive fusion module (LPFM), which could improve the reconstruction accuracy while achieving high computational efficiency.

### 3.2. Perceptual Quality Methods

The PSNR and SSIM are two important metrics commonly used for image quality assessment judgment. Recently, Hsu et al. [79] proposed the Dense Residual Connected Transformer (DRCT), which has achieved good results in achieving a high PSNR and SSIM. Blau et al. [80] first revealed the existence of perceptual distortion trade-offs. Subsequently, Xue et al. [81] proposed the unreachable area as a new indicator for evaluating and comparing the trade-off between distortion and perception, achieving better results. However, the question of how to improve the perceptual quality while avoiding distortion still needs to be addressed. Therefore, this section proposes several methods to address this trade-off dilemma, aiming to achieve the dual goals of reducing image distortion and maintaining good perceptual quality.

Perceptual Loss: Although pixel-based losses like the L1 loss (mean absolute error) and L2 loss (mean square error) have been heavily utilized in the pursuit of high-quality images, the loss function focuses only on pixel matching, without paying attention to the visual quality of the reconstructed image, and it is usually difficult to capture the differences between SR images and high-resolution images at the perceptual level. Therefore, to address this issue, methods for content loss, texture loss, object perception loss, dual perception loss, and wavelet domain loss have been proposed, and these methods enable the loss function to more accurately reflect the perceptual and semantic differences between images.

Content loss: This involves ensuring that the reconstructed image is highly consistent with the target image in terms of content. For example, aligning SR images with human visual perception features can be used to integrate content loss metrics into SISR, and a mechanism for content loss can be implemented through pretrained classification networks. Generally speaking, pretrained classification networks such as VGG [82] and ResNet [25] are the most used. By employing this framework, the semantic discrepancies across visual representations can be precisely quantified. The content loss can be expressed as follows:

(16) $$\begin{array}{c} L_{\mathrm{content}\;}=\frac{1}{H_{l}W_{l}C_{l}}\sum_{i=1}^{H_{l}}\sum_{j=1}^{W_{l}}\sum_{k=1}^{C_{l}}\left|\phi_{l}\left(I_{SR}\right)-\phi_{l}\left(I_{HR}\right)\right| \end{array}$$

where $\phi_{l}$ is the feature extractor of the *l*th layer of the pretrained network. $H_{l}$, $W_{l}$, and $C_{l}$ represent the height, width, and the number of channels of the feature map in the *l*th layer.

Texture loss: Texture loss is also known as the style reconstruction loss. Gatys et al. [83,84] were inspired by the transfer of artistic styles in oil painting and proposed the texture loss for SISR. In SISR, the core goal is to reconstruct images with consistent texture, color, and edge features, which means that the reconstructed image should be highly consistent with the target image in terms of style. Assuming that the target image is It and the generated image is Ig, their Gram matrices are G (It) and G (Ig), respectively. Texture loss is typically calculated using the mean square error (MSE), where C is the dimension of the Gram matrix, i.e., the number of channels in the feature map. The calculation formula is

(17) $$\begin{array}{c} L_{\mathrm{texture}}=\frac{1}{C^{2}}\sum_{i=1}^{C}\sum_{j=1}^{C}{\left(G{\left(I_{t}\right)}_{i,j}-G{\left(I_{g}\right)}_{i,j}\right)}^{2} \end{array}$$

With the texture loss, the model-generated image is closer to the target image in terms of texture details, which improves the image quality, reduces artifacts, and enhances the visual fidelity and model generalization [85].

Targeted perceptual loss: The traditional perceptual loss has limited capabilities due to the fact that semantic information is not taken into account in the image reconstruction error. Rad et al. [86] proposed a new approach by optimizing a deep network-based decoder using a targeted objective function that divides the image into three regions, namely the background (gb), the boundary (ge), and the target (go), and using the segmentation from the labels generated from the object, background, and boundary (OBB) labels to estimate the appropriate perceptual loss of the boundary while considering the texture similarity of the background, resulting in more realistic textures and sharper edges. Guo et al. [87] used targeted perceptual loss to guide the network to recover the texture details of the image; compared to SRGAN and other networks, their method can generate more realistic and reliable textures.

Double perception loss: Abnormal feature propagation in the decoder module can exacerbate model instability due to excessive scaling operations. This mechanism will continuously distort and accumulate in the network layer, systematically compromising the authenticity of the output image. Song et al. [88] proposed the dual perceptual loss, which takes into account the benefits of learning two features simultaneously and effectively enhances image reconstruction.

Wavelet domain loss: In the current methods, attempting to reconstruct image details can result in artifacts and illusions. Korkmaz et al. [89] showed that, by training the SR model of the GAN using a wavelet domain loss function, it is possible to better learn the features of real details versus artifacts. Extensive experimental results show that the model achieves a better perception–distortion trade-off based on multiple objective measurements and visual assessments.

#### 3.2.1. Adversarial Training

**Figure 11:**
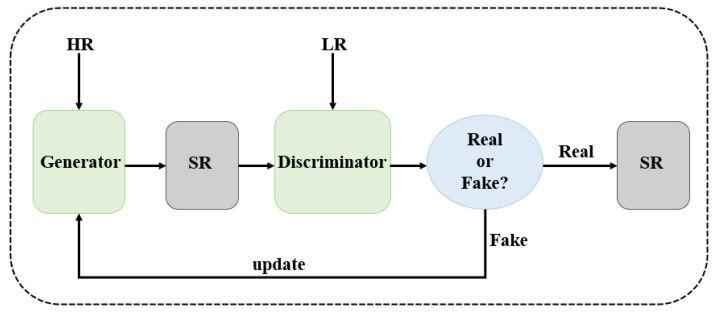
Generative adversarial networks.

The generative adversarial network is a new framework for evaluating generative models through an adversarial process, proposed by Goodfellow et al. [20] in 2014. As shown in Figure 11, the framework mainly consists of a generator for capturing the data distribution and a discriminator for judging the “authenticity” of the input data, and the framework trains the network by means of adversarial games.

Ledig et al. [21] first applied generative adversarial networks (GANs) to SR images and proposed the Super-Resolution Generative Adversarial Network (SRGAN). SRGAN not only utilizes a GAN to improve the network structure but also applies perceptual loss to SR and adopts a loss function that combines perceptual loss and adversarial loss, significantly improving the details of reconstructed images in terms of perceptual quality. However, the “deception” of the GAN framework results in relatively low objective evaluation metrics, such as the PSNR and SSIM. Inspired by SRGAN, Wang et al. [90] proposed ESRGAN, which achieves more effective perception reconstruction by improving SRGAN. Wang et al. [91] proposed a new GAN inverse method that utilizes the powerful generation abilities of StyleGAN-XL while improving the quantitative and qualitative results of SISR. In 2024, Yang et al. [92] improved the generator of SRGAN and designed a multi-scale feature fusion module as the backbone feature extraction network of the generator. This module represents the multi-scale features of images at the granularity level and extends the perceptual domain of each network layer, enabling the full extraction of image feature information. This model enhances texture details and achieves better visualization effects. Huang et al. [93] proposed a network architecture, CL-GAN, which extracts features from clustered image regions and constructs them based on the GAN framework. This architecture enhances the SR reconstruction process and improves the image reconstruction performance.

#### 3.2.2. Cycle Consistency

The theory of cyclic consistency assumes that there is a mapping relationship between the source domain and the target domain. Zhu et al. [19] proposed a cyclic consistency mechanism. In the absence of paired examples, they propose a learning method that can convert images from the source domain *X* to the target domain *Y*. The goal of this method is to use adversarial loss to learn a mapping *G*: *X*→*Y*, so that the distribution of images generated by G(X) is the same as that of *Y*. This mapping is extremely underconstrained; thus, the approach relates it to the inverse mapping F: *Y*→*X* and pushes F(G(X))≈X by introducing the cyclic consistency loss. The superiority of this approach has been shown by quantitative comparisons with several earlier approaches.

In SISR, the idea of cyclic consistency has also received a lot of attention. We can learn both the mapping from LR to HR and the inverse procedure given an LR image domain X and an HR image domain Y. It has been demonstrated by researchers that more realistic images can be achieved by learning how to perform image degradation without paired data [94]. A cell cycle network is suggested in CinCGAN [95] that upsamples using a pretrained model after first mapping noisy and ambiguous inputs to the noiseless LR domain. This method can improve the quality and related performance of image reconstruction. In DRN [96], a closed loop that offers extra supervision is formed by learning a mapping from HR images to LR images to estimate the downsampling kernel and reconstruct the LR images. In addition, DRN provides us with a new learning method that can directly learn from LR images, making the images closer to the real world. In SGA [97], the correlation between pixel features is constructed through a parameter-free Gram matrix, and the invariance of the feature layer is maintained by using the consistency loss. This loss normalizes the consistency between the LR input and its corresponding LR output, which enables us to reconstruct more realistic images and improves the correlation performance.

#### 3.2.3. Diffusion-Based Methods

In the field of SISR, diffusion-based methods have received extensive attention in recent years, inspired by the Denoising Diffusion Probabilistic Model (DDPM). To date, a series of representative models have emerged in the field of diffusion-based methods.

SRDiff [98] is the first model to apply diffusion models to SISR. It utilizes the iterative properties of Markov chains and uses LR as input to gradually convert Gaussian noise into HR. This process enables the diversification and realism of SISR, laying an important foundation for subsequent research. Regarding SR3 [99], its uniqueness lies in its training method, which trains the model at different levels of noise, enabling the model to be optimized for Gaussian noise input. Compared with GANs, SR3 generates images that are more realistic and natural, and it can present more delicate and vivid image details. Meanwhile, it is worth noting that SR3 adopts a multi-stage approach and performs processes such as scaling optimization, reflecting the concept of hierarchical diffusion. Layered diffusion has significant advantages in efficiency. It can ensure the rationality of the global structure while improving the authenticity of local details, effectively enhancing the reconstruction efficiency. In IDM [100], the framework innovatively integrates implicit neural representations with denoising diffusion models in an end-to-end manner. This helps the model to learn continuous image resolution representations, enabling it to generate images of different resolutions and further expanding the application scope of diffusion-based methods. DR2 [101] mainly focuses on processing complex low-quality facial images. The algorithm first uses DDPM to denoise and extract features from low-quality facial images. Then, through a designed enhancement module, the preliminary processed images are optimized and reconstructed, ultimately restoring them to high-resolution and clear facial images. This has important application value in related fields such as facial recognition.

At present, there are still some methods that utilize existing diffusion-based models to assist SISR in improving the accuracy of related reconstruction. StableSR [102] and DiffBIR [103] are two models that utilize the powerful prior knowledge of pretrained text-to-image diffusion models. By fine-tuning these pretrained models, StableSR and DiffBIR can effectively combine text information with the image SR task, achieving good results in real-world SISR and providing new ideas for the resolution of image SR problems in practical scenarios. Similarly, the integration of pretrained models and SISR demonstrates enormous potential. Pretrained models can provide guidance for SISR by learning image semantics and other information from large amounts of data, thereby accelerating the speed and improving the accuracy of image reconstruction. For example, by aligning the features of LR input with the semantic embeddings of pretrained models, SISR models can better maintain consistency in the object shape and scene structure during upsampling. DiffIR [104] utilizes a model pretrained on real images and incorporates rich prior information into the SISR model. This approach enables DiffIR to more accurately capture the features and structures of the image, greatly improving the efficiency and accuracy of the model. Wu et al. [105] proposed the potential diffusion of continuous-scale super-resolution (LDCSR) model. This model adopts a two-stage latent diffusion paradigm to capture the prior differences between HR and LR images and learns in the latent space to predict the true prior differences, thereby improving the quality of image reconstruction.

Although diffusion-based SISR methods have achieved remarkable results in terms of image generation fidelity, effectively reducing artifacts and generating more realistic and natural images, they also face some challenges. On the one hand, this type of method requires a large number of new samples for training to ensure that the model can learn sufficient image features and variation patterns; this may be limited by data acquisition and storage issues in practical applications [106]. On the other hand, the convergence speed of the model is slow, meaning that it takes a relatively long time to process images, making it difficult to meet the requirements of some applications with high real-time requirements. Recently, the question of how to overcome these shortcomings and further improve the efficiency and practicality of diffusion-based methods has become one of the key research areas for researchers in this field.

### 3.3. Additional Information Utilization Methods

We have already introduced the relevant work on improving the image perception quality in the above. In this section, we will explore how to utilize the additional information of images to enhance the model’s correlation performance, enabling the model to better reconstruct real images.

To further enhance the performance of models, researchers have explored various additional information utilization methods. These methods leverage various types of information both inside and outside the image, providing new ideas and approaches for the optimization of SISR models. These methods are mainly based on fundamental methods such as image internal statistics, multi-factor learning, prior guidance, reference images, and knowledge distillation.

Methods Based on Image Internal Statistics: Zontak et al. [107] discovered that there are some image patches in images for which it is difficult to find matches in external databases. Moreover, the internal entropy of image patches within a single image is much smaller than the external entropy of patches in a collection of natural images. Based on this characteristic, methods that utilize image internal statistics show unique advantages when dealing with such images. Take the ZSSR [18] method as an example. It fully utilizes the internal statistical properties of the image to train a specific CNN for that image. During the training phase, multiple LR-HR image pairs are generated through data augmentation techniques and used to train the CNN. In the testing phase, the LR images are input into the trained CNN to obtain the reconstructed HR image. In SinGAN [108], a fully convolutional GAN with a pyramid structure is constructed to learn the internal block distributions of images at different scales. When generating HR images, multiple upsampling operations are performed on LR images to obtain an SR output.

Multi-Factor Learning Methods: Traditionally, SISR usually requires the training of specific models for different upsampling factors, which undoubtedly increases the complexity and cost of model training. The multi-factor learning method can overcome this limitation by deeply exploring the scale correlations among various upsampling factors, allowing a single model to adapt to multiple different upsampling factors and thus enhancing the model’s performance. In LapSRN [40], the LR image is gradually reconstructed in a pyramid network structure. During this process, not only can the final large-scale reconstruction result be obtained, but also the intermediate results can be directly used as the outputs corresponding to the respective multiple factors. Wang et al. [109] also found that there are correlations among multi-scale tasks. Based on this, they designed specific scale processing modules at the beginning and end of the model to handle different upsampling factors. Li et al. [45] further optimized this strategy in the MDCN model. Compared with MDSR, MDCN can effectively utilize model parameters and learn correlations between different scales to a greater extent. Optimizing this strategy can enhance the effectiveness of the model, thereby improving the related performance.

Prior Guidance Methods: Currently, many SISR methods tend to build end-to-end CNN models to complete SR tasks. However, in the process of image processing, due to the loss or damage of many useful image features, these models are not satisfactory in reconstructing real high-frequency details. To address this issue, the SISR framework based on prior knowledge has emerged. Many experiments have shown that, by utilizing prior image information, models can not only converge faster but also achieve higher reconstruction accuracy. In recent years, various types of image priors have emerged, such as total variation (TV) priors, sparse priors, and edge priors. Yang et al. [110] proposed a Deep Edge-Guided Recursive Residual (DEGREE) network for SR image reconstruction. Fang et al. [111] proposed an efficient and accurate soft edge-assisted network (SeaNet). Compared to DEGREE, SeaNet can learn more accurate image edges. Moreover, image prior methods also play a positive role. For example, in SFTGAN [112], a spatial feature transformation (SFT) technique is used to reconstruct more realistic image details through semantic category priors. In SPSR [113], the model utilizes gradient maps and integrates them into the SR branch to provide better prior information, which facilitates image reconstruction, improves image authenticity, and enhances texture details. In FeMaSR [114], VQ-GAN [115] is first pretrained and its related discrete features are provided as prior information to the model, which can effectively achieve high-quality image restoration. Huang et al. [116] proposed the Multi-Modal Prior-Guided Diffusion Model (MPGSR), which involves two stages of multi-modal prior-guided extraction and adaptive guided injection and can restore realistic high-resolution images with better performance.

Reference Image Methods: Unlike traditional SR methods, reference image-based SISR (RefSR) uses additional reference images to assist SR in reconstruction. Meanwhile, in order to enhance the texture information of images, researchers have developed various methods, such as image registration and patch matching. Some RefSR methods [117,118] have been proposed, such as those assuming that the reference image and LR image have similar content and aligning these two images. For example, Yue et al. [117] were able to minimize the resource consumption by matching reference images with LR images. In CrossNet [118], it is proposed to use optical flow technology to align the reference image and the LR image at different scales and connect the aligned images to the corresponding layers of the decoder. Zhang et al. [119] applied patch matching between LR images and reference images to achieve the adaptive transfer of the reference image texture to LR images. In TTSR [120], Yang et al. proposed a texture transformation network that can extract corresponding texture details from images and transfer them to LR images, thereby improving the image quality. Cong et al. [121] proposed reference-based iterative interaction (RIISSR) for stereo image super-resolution, which mainly utilizes P2 matching to establish cross-correspondence relationships, demonstrating better performance.

Knowledge Distillation Methods: Knowledge distillation is a technique that transfers the representational power of large models to small models, and it has shown significant effectiveness in many computer vision tasks. Knowledge distillation is mainly divided into two types, namely soft label distillation and feature distillation. Regarding soft label distillation, the softmax output of large models serves as soft labels, providing valuable implicit knowledge for small models. Regarding feature distillation, the intermediate feature maps of large models are transferred to small models for easier extraction. In the field of SISR, some studies have introduced the knowledge distillation technique to further improve the performance of lightweight models. In SRKD [122], a small network is guided by a larger network, and it can achieve similar feature distributions to a large-scale network. JDSR [123] explores joint distillation by combining relevant information, effectively improving the performance of lightweight models. CSD [124] innovatively combines contrastive learning and distillation tasks to further resolve the related problems of lightweight models and optimize model performance. Romina et al. [77] proposed MambaLiteSR, which utilizes the Vision Mamba architecture, integrates state space blocks and reconstruction modules, and employs knowledge distillation to optimize the performance and improve the efficiency.

### 3.4. Comparison of Method Types for Simulating SISR

Based on the SISR technology classification framework, including efficient network design, perceptual quality optimization, and additional information utilization, we list three types of SISR models in Table 3. From the PSNR and SSIM indicators in the table, it can be seen that partitioned SISR technology has a significant performance improvement. Therefore, the proposed classification framework can effectively classify SR methods and reflect their performance characteristics, achieving progress compared to previous work.

### 3.5. Real-World Image SR

In real-world scenarios, image degradation patterns are complex and difficult to model, often involving anisotropic blur, downsampling, and the addition of signal-dependent noise. Additionally, images are affected by the in-camera signal processing (ISP) pipeline. These factors result in the poor performance of traditional SISR models when applied to real-world images. Furthermore, most existing models are restricted to specific integer upscaling factors, significantly limiting their practical application and generalizability. To address these challenges, researchers have proposed a series of deep learning-based methods, which can be broadly categorized into two main approaches: blind image SR and arbitrary-scale SR.

#### 3.5.1. Blind Image SR

Blind SISR, which aims to perform SR reconstruction on LR images corrupted by unknown and complex degradation processes, has attracted considerable interest due to its critical role in real-world applications. However, it is important to note that there is no standardized definition for blind SISR. For a comprehensive discussion of its scope and challenges, readers may refer to [126]. In this study, we adopt a simplified taxonomy for blind SISR methodologies, dividing them into two primary categories according to their degradation modeling strategies: explicit degradation modeling and implicit degradation modeling.

Explicit Degradation Modeling: With the advancement of deep learning technologies, significant progress has been made in the field of blind SISR. Explicit degradation modeling, as a critical component, provides essential support for high-quality image restoration. Early methods had certain limitations, such as the internal GAN-based kernel estimation method proposed by Bell Kligler et al. [127], which overly relied on kernel estimation modules and lacked unified modeling. To address this limitation, researchers have begun exploring methods to integrate degradation estimation and SR reconstruction into a unified framework. For instance, Gu et al. [128] proposed an iterative kernel correction algorithm that progressively refines blur kernel estimates through alternating optimization, while Luo et al. [129] introduced the deep alternating network (DAN), which simultaneously estimates blur kernels and reconstructs SR images within a single network. These methods significantly improve model robustness and the reconstruction quality but still face challenges in complex degradation scenarios, where estimation errors could lead to reconstruction failures.

In recent years, deep learning-based integrated approaches for degradation estimation and SR reconstruction have become a research hotspot. Sun et al. [130] proposed the learning correction error (LCE) method, which uses a lightweight corrector to generate corrected LR images, reducing the space required and improving the correlation performance. Li et al. [131] introduced BlindDiff, which combines the maximum a posteriori (MAP) optimization framework into the diffusion model to reconstruct SR images and make them clearer. Ohayon et al. [132] approached image restoration from the perspective of fairness, proposed the concept of perceived fairness (PF), and defined a group perception index (GPI) to evaluate the restoration quality between different images. Additional frameworks include IKR-Net [133], which handles complex degradation through iterative kernel reconstruction and noise estimation, and CDFormer [134], which utilizes content-aware degradation-driven Transformers to capture degradation and content representation, further advancing blind image SR technology.

Implicit Degradation Modeling: In blind SISR research, implicit modeling has received a lot of attention, and it mainly guides SR reconstruction through learning image degradation features. In 2021, Wang et al. [135] proposed DASR, a method based on self-supervised contrastive learning that compares the features of different image blocks through contrastive learning. DASR implicitly captures degraded information without explicit parameter estimation, and it uses degraded features to guide the SR network for high-quality reconstruction under unknown degradation. Subsequently, Yang et al. [136] proposed DMSR in 2022, which is a method that utilizes the degradation of BlindSR to guide the network and improve the quality of image restoration.

Furthermore, Chen et al. [137] introduced DIEM, a dynamic degradation intensity estimation module for adaptive BlindSR. DIEM dynamically estimates pixel-level degradation information to guide reconstruction, and its core innovation is that it generates specific degradation feature maps for input changes, effectively solving common problems such as oversmoothing or oversharpening in real-world scenarios. Liu et al. [138] proposed a blind SR model based on Transformer in 2024. The model integrates contrastive learning into the Transformer network and combines the advantages of a CNN and Transformer to extract local features and capture global semantic features. The model has achieved significant performance improvements on multiple benchmark datasets. Jiang et al. [139] proposed a blind image super-resolution framework (CdCL) based on content-decoupled contrastive learning and designed an implicit degradation-adaptive module for detail perception. This framework and module can better adapt degraded representations to LR features, reduce model complexity, and improve model performance. In summary, implicit modeling in blind SR has undergone significant evolution, and future research may focus on optimizing relevant degradation feature learning and exploring more complex degradation scenarios to enhance model generalization and robustness.

#### 3.5.2. Arbitrary-Scale SR

Despite the continuous development of SISR technology, it still faces many challenges in practical applications, such as using a single model to handle arbitrary scaling factors. To address these issues, Wang et al. [140] proposed a multi-scale dilated convolution residual network, which includes residual attention dense blocks (RADBs) and dilated multi-scale residual modules (DMRMs). The RADB is used to learn features from the original low-resolution images, enhancing the network’s ability to recognize both high- and low-frequency information. The DMRM, based on dilated convolutions, aims to address the limitations of the convolutional receptive field and potential information loss when extracting features through a single channel. However, despite the excellent performance of these methods at specific scales, they still have limitations when dealing with arbitrary scale factors. To overcome this, Li et al. [141] proposed a novel multi-scale cross-fusion network (MCNet) for efficient SR tasks with arbitrary scale factors. MCNet introduces a scale-wise module (SWM) and a multi-scale cross-fusion module (MSCF), which combine scale information with pixel features and enrich spatial information through multi-scale feature interaction learning while removing redundant noise. Wei et al. [142] proposed a dynamic implicit network (DINet) for multi-contrast MRI with arbitrary-scale SR, which mainly includes scale-adaptive dynamic convolution, a dual-branch implicit attention module, and the fusion of response modules to improve model performance.

### 3.6. Domain-Specific Applications

**Figure 12:**
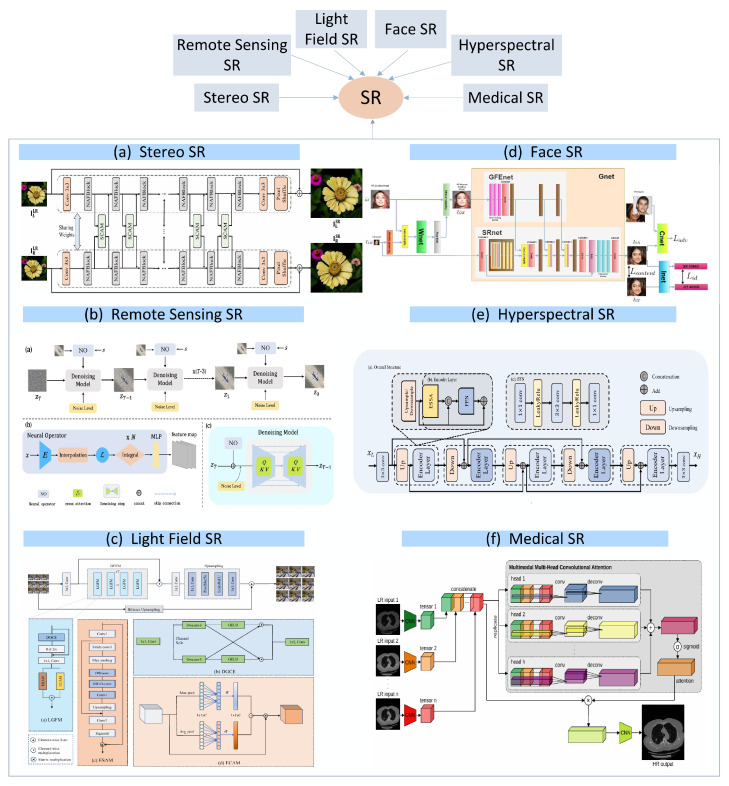
Examples of various popular SR tasks. (**a**) NAFSSR [143] for stereo SR, (**b**) NeurOp-Diff [144] for remote sensing SR, (**c**) LGFN [145] for LF SR, (**d**) GWAInet [137] for face SR, (**e**) Essaformer [146] for hyperspectral SR, and (**f**) MMHCA [147] for medical SR.

At present, image SR technology has been widely applied in various fields. This section will introduce various application scenarios for SR technology, as shown in Figure 12.

#### 3.6.1. Stereo Image SR

As is well known, dual-camera systems are commonly used for depth information estimation, but stereo imaging technology can also play a role in image restoration tasks. In these tasks, it is necessary to process two images with a difference of more than one pixel, and fully utilizing the information of these two images can effectively improve the spatial resolution.

In StereoSR [148], Jeon et al. proposed a method to improve the resolution of stereo images by learning the sub-pixel disparity. However, in this method, there are still certain challenges when dealing with large stereoscopic images with significant disparity variations. To address this issue, Wang et al. [149,150] proposed a disparity attention mechanism with a global receptive field along the epipolar line, which can generate reliable correspondences between stereo image pairs and thus improve the quality of the reconstructed SR images. In [151], the researchers introduced a high-quality stereo image dataset named Flickr1024, which contains 1024 pairs of stereo images, providing a rich resource for stereo image SR research. In [152], the authors designed a stereo attention module that integrates stereo information with a pretrained single-image SR network through bidirectional interaction, further enhancing the performance of stereo image SR. In [109], a symmetric bidirectional disparity attention module was proposed, combined with an inline occlusion handling scheme, to achieve effective cross-view information interaction. In [153], the researchers proposed SSRDE-FNet, a unified framework capable of simultaneously handling stereo image SR and disparity estimation tasks. In [143], in addition to using NAFNet [154] to extract single-image features from the left and right views separately, a stereo cross-attention module was introduced to fuse features from both views. Furthermore, in [155], the researchers proposed Steformer, an efficient Transformer-based stereo image SR model. Through the residual cross Steformer block (RCSB) and cross-to-internal attention mechanism (C2IAM), Steformer demonstrates excellent performance in cross-view information extraction and feature fusion. In [156], the double stereo cross-attention module (DSCAM) was proposed, which enhances the integration of complementary cross-view information through an overlapping window mechanism and refines and emphasizes the combined features through additional multiplication steps.

#### 3.6.2. Remote Sensing Image SR

At present, satellite image processing technology is constantly advancing, and remote sensing is also playing an increasingly important role. However, remote sensing technology has significant shortcomings in terms of spatial resolution, spectral resolution, and radiometric resolution. Therefore, the current remote sensing technology still faces many problems.

At present, there are various innovative remote sensing image SR methods. For example, in [157], the researchers proposed an innovative unsupervised hourglass neural network architecture that can be used to enhance the SR effects of remote sensing images. This network introduces diverse spatial patterns by generating random noise, which can be extended under global reconstruction constraints to improve the image quality. In [158], the researchers designed a deep residual compression and excitation network (DRSEN) to address the complex distribution of remote sensing images. In [159], a mixed high-order attention network (MHAN) was proposed, which consists of two parts: one is a feature extraction network for the efficient extraction of image features, and the other is a feature refinement network with a high-order attention mechanism dedicated to detail recovery to enhance the SR effects of images. In [160], a Dense Sampling Super-Resolution (DSSR) network was proposed to explore large-scale remote sensing image SR reconstruction, significantly improving the image resolution through an optimized network structure and sampling strategy. In [161], a hybrid-scale self-similarity development network (HSENet) was proposed, which can utilize both single-scale and cross-scale similarities to achieve high-quality image reconstruction. In [162], Wang et al. proposed a multi-scale enhancement network (MEN) that fully utilizes the multi-scale features of remote sensing images to significantly enhance the network’s reconstruction capabilities. In [163], Liu et al. proposed a dual-learning-based graph neural network (DLGNN), in which the graph neural network (GNN) aggregates adjacent feature patches across scales, fully considering the self-similarity of remote sensing images to further enhance SR effects. In [144], a continuous remote sensing image SR method was proposed, which is based on the neural operator diffusion model (NeurOp-Diff). This method learns arbitrary-scale resolution representations through neural operators and uses diffusion models for denoising, effectively solving the problems of artifacts and oversmoothing in existing SR methods. In [164], a new framework called ESC-MISR was proposed for the multi-image super-resolution (MISR-RS) task in remote sensing, which reconstructs high-resolution images by enhancing the spatial correlations between multiple images.

#### 3.6.3. Light-Field Image SR

A light-field (LF) camera is a device with unique advantages that can capture the light-field information emitted by the scene and has multiple related views. In recent years, its importance has been constantly highlighted. However, LF cameras face a trade-off between spatial and angular resolutions [165]. To overcome this limitation, SR technology was introduced. In [166], the researchers introduced a cascaded convolutional neural network architecture to simultaneously enhance the spatial and angular resolutions of light-field images. They also constructed a new light-field image dataset for training and validation. In order to reduce the dependence on precise depth or disparity information as a prior for LF image SR, Sun et al. [167] proposed a bidirectional recurrent convolutional neural network and an implicit multi-scale fusion strategy for SR image reconstruction. In [168], a Transformer-based LF image SR method was proposed, which integrates complementary information between different views using corner Transformers and captures local and long-range dependencies in each sub-aperture image using spatial Transformers. In [145], a lightweight LF image SR method was proposed, which introduces a local convolutional modulation module to achieve efficient feature extraction and fusion, enabling the model to capture more detailed information in the light-field image while maintaining a lightweight design. Wang et al. [169] proposed a practical SR method for real light-field images. This convolutional neural network, designed by learning to adjust to different degradations and integrating spatial and angular information from the light field, achieves better performance and generalization to real light-field images.

#### 3.6.4. Face Image SR

Among the many directions of SR technology applied to domain-specific images, face image SR is the most well known. Due to its huge application potential in facial recognition systems, such as security and surveillance systems, face image SR has developed into a research hotspot, attracting numerous scholars. In recent years, facial image SR has been continuously improving. In [170], CPGAN was proposed, which optimizes image quality through traditional facial SR loss and a new illumination compensation loss. In [171], the generator architecture adopts a spatial Transformer network to overcome problems related to the misalignment of input images. In [172,173], it is shown that minimizing the distance between SR and HR facial images can preserve identity-related features and improve the image accuracy. In [174], mask occlusion is regarded as image noise, and a joint and collaborative learning network (JDSR-GAN) is constructed to handle the SR task for masked face images. Qiu et al. [175] proposed a dual-path hybrid network (DPHNet) that can solve the SR problem of motion-blurred facial images. The network includes CNN and Transformer branches, which are integrated through convolutional block attention modules to effectively improve the performance. These methods [175,176,177,178] are mainly based on the prior generation of a GAN, which can restore the image to a realistic, textured, and high-definition facial image.

#### 3.6.5. Hyperspectral Image SR

Unlike the human eye, which can only perceive visible light, hyperspectral imaging technology has the ability to collect and process information in the full electromagnetic spectrum range [179]. However, the performance of hyperspectral systems has certain limitations, and it is difficult to balance the spatial resolution and spectral resolution. Based on this, researchers have conducted research on hyperspectral image SR to overcome this difficulty.

In [180], multi-scale 3D convolution was used to capture local features in both the spectral and spatial domains. By integrating the advantages of the Mamba model in efficiently modeling long-distance information, good results have been achieved. In [146], Zhang et al. proposed an efficient Transformer model for hyperspectral image SR through a novel and efficient self-attention method based on the SCC kernel. In [181], a spectral and correlation algorithm for hyperspectral remote sensing image SR was introduced, which can fully represent the relevant features, improve the efficiency, reduce the costs, and thus improve the spatial resolution of hyperspectral images. In [182], Dang et al. proposed a dual-branch convolutional neural network for the fusion-based SR task of hyperspectral images. This neural network is based on cross-modal feature attention interaction and can generate hyperspectral images with high-fidelity spectral and spatial information. In [183], a hyperspectral image SR method based on adaptive depth prior regularization is adopted, which can solve the problem of the excessive dependence on priors in current hyperspectral SR methods, resulting in improved parameter interpretability. Jia et al. [184] proposed DiffHSR, which utilizes multi-modal techniques and generative priors and employs low-cost data and fine-tuning methods to more effectively improve the processing of different degraded images and enhance the image quality.

#### 3.6.6. Medical Image SR

Medical imaging SR plays an irreplaceable role in clinical diagnosis and surgical planning, as well as in medical imaging, such as computed tomography (CT) and magnetic resonance imaging (MRI). Clear and accurate images can help medical staff to comprehensively understand the patient’s physical condition, provide strong support for subsequent medical decisions, and facilitate accurate disease diagnosis and reasonable surgical plans. The rapid development of deep learning technology has brought new opportunities to the field of medical image SR.

Chen et al. [185] proposed a multi-level dense connected super-resolution network (mDCSRN) trained under GAN guidance, which can not only be used to generate HRMR images but also accelerate the computation speed. In [186], a 3D super-resolution convolutional neural network (3DSRCNN) was proposed, which can improve the resolution of 3D-CT volumetric images and enhance the image quality. In [187], in order to reduce the burden of model representation, Zhao et al. proposed a deep channel segmentation network (CSN). In [188], Peng et al. introduced a spatially aware interpolation network (SAINT) for medical slice synthesis, which can effectively alleviate the storage limitations of voxel data. In [189], Feng et al. proposed a Task Transformer Network (T2Net) that can share representations and transfer features between reconstruction and SR tasks. In [147], Georgescu et al. used multi-modal LR input for medical image SR and proposed a novel multi-modal multi-head convolutional attention mechanism for multi-contrast medical image SR. In [190], implicit neural network representation (INR) was proposed, which is an emerging deep learning technique that overcomes the limitations of discrete voxel grids by representing medical images as continuous functions. It has demonstrated its efficiency and flexibility in medical image reconstruction tasks. Kui et al. [191] proposed the Iterative Collaborative Network (ICONet), which mainly consists of a reconstruction branch, an SR branch, and an SR-Rec fusion module. It can effectively solve the problems of incomplete feature extraction in traditional medical image SR methods and insufficient interaction between tasks in multi-task learning. Li et al. [192] proposed a self-correcting texture supplementation network (STS-SR) for reference-based image super-resolution. This network includes a texture-specified self-correcting feature transfer module and a cross-scale texture complementary network; it performs well and can improve the role of autonomous artificial intelligence in healthcare.

## 4. Reconstruction Results

In this section, we list some representative reconstruction results obtained from SISR models and compare them, including the training dataset and model parameters, as shown in Table 4. Based on the differences in relevant data, it can be found that (1) for the model, large datasets can produce better reconstruction results and better correlation performance; (2) contrary to traditional belief, a model’s performance might not improve simply if it has more parameters; (3) Transformer-based models can demonstrate unique advantages, thereby achieving better model performance; (4) the research on micro-models is still not advanced, and, in the future, we should focus on conducting research in this area to overcome the difficulties faced by micro-models.

Furthermore, it is worth noting that excellent SISR results play a crucial role in improving object detection and classification performance. In object detection scenarios, SISR reconstruction can restore the subtle details of the target object during the detection process, thereby improving the detection accuracy. For example, in YOLO-SASE [193], the algorithm combines the YOLO detection framework with the SRGAN network, which can improve the detection ability for infrared small targets in complex backgrounds. Similarly, in image classification, due to the low resolution and blurry images typically obtained, SISR processing can provide clearer texture and boundary information, thereby improving the accuracy of classification.

**Table 4:** Model comparison.

Model	Set5 PSNR/SSIM	Set14 PSNR/SSIM	Urban100 PSNR/SSIM	MOS	Training Datasets	Parameters
SRCNN [7]	30.48/0.8628	27.50/0.7513	24.52/0.7221	3.2	T91+ImageNet	57 K
VDSR [26]	31.35/0.8838	28.02/0.7680	25.18/0.7540	3.5	BSD+T91	665 K
LapSRN [40]	31.54/0.8855	28.19/0.7720	25.21/0.7560	3.6	BSD+T91	812 K
MemNet [34]	31.74/0.8893	28.26/0.7723	25.50/0.7630	3.7	BSD+T91	677 K
IDN [59]	31.82/0.8903	28.25/0.7730	25.41/0.7632	3.7	BSD+T91	678 K
RFDN [60]	32.18/0.8948	28.58/0.7812	26.04/0.7848	3.8	DIV2K	441 K
DSRN [37]	31.40/0.8830	28.07/0.7700	25.08/0.7470	3.4	T91	1.2 M
MSRN [29]	32.07/0.8903	28.60/0.7751	26.04/0.7896	3.9	DIV2K	6.3 M
CARN [38]	32.13/0.8937	28.60/0.7806	26.07/0.7837	4.0	BSD+T91+DIV2K	1.6 M
SeaNet [111]	32.33/0.8970	28.81/0.7855	26.32/0.7942	4.1	DIV2K	7.4 M
CRN [38]	32.34/0.8971	28.74/0.7855	26.44/0.7967	4.1	DIV2K	9.5 M
EDSR [27]	32.46/0.8968	28.80/0.7876	26.64/0.8033	4.2	DIV2K	43 M
RDN [194]	32.47/0.8990	28.81/0.7871	26.61/0.8028	4.2	DIV2K	22.6 M
MDCN [45]	32.48/0.8985	28.83/0.7879	26.69/0.8049	4.3	DIV2K	4.5 M
SRRFN [39]	32.56/0.8993	28.86/0.7882	26.78/0.8071	4.3	DIV2K	4.2 M
RCAN [48]	32.63/0.9002	28.87/0.7889	26.82/0.8087	4.4	DIV2K	16 M
SwinIR [69]	32.92/0.9044	29.09/0.7950	27.45/0.8254	4.5	DIV2K+Flickr2K	11.8 M

## 5. Potential Issues and Future Directions

### 5.1. Lightweight SISR for Mobile Intelligence

At present, with the increasing demand for smart devices, the requirements for lightweight SISR models are also becoming higher, which makes related research crucial. Although there has been some progress in current lightweight models, due to limitations such as computational costs, these models still cannot meet the requirements of some mobile intelligent devices and edge sensors in terms of size and performance. Therefore, studying lightweight SISR models not only plays an important role in promoting their development but also facilitates their commercial application in sensor scenarios.

To achieve this goal, further exploration of more efficient network architectures and mechanisms is needed. It is worth noting that evaluating the hardware adaptability of these lightweight models on edge devices is also crucial, as they are commonly used in sensor scenarios, such as the NVIDIA Jetson Nano and Arduino. For classic embedded AI platforms such as the NVIDIA Jetson Nano, the hardware memory limit is 4 GB, and key metrics evaluated include the inference latency and memory usage. For the Arduino, its hardware resources are extremely limited, e.g., the Arduino Uno only has 32 KB of RAM. When evaluating the inference latency, an ultra-lightweight architecture is required. In terms of the Arduino’s memory usage, its model parameters should be compressed to the KB level, and large-scale feature maps should be avoided during inference. We calculate the costs on two platforms, as listed in Table 5.

In addition, when studying SISR models, the model can be combined with compression techniques such as network binarization and network quantization to balance relevant performance aspects while meeting size requirements.

Overall, by integrating hardware evaluation into the optimization of lightweight SISR models, edge sensor devices can be better matched, providing better applicability for lightweight devices.

### 5.2. SISR Applicable to Multiple Scenarios

Through the existing DL-based SISR models, it can be seen that the relevant research on these models is relatively mature. However, in-depth research on these models reveals certain limitations. For example, during the training and testing phases, these models need to maintain consistent related structures, but this requirement limits their applicability, making it difficult for a single model to be widely applicable to various types of scenarios. This limitation requires the design of specialized models for different scenarios, but this often increases the costs (including manpower and material resources). To address this deficiency, it is essential to design a flexible SISR model. At the same time, when designing such SISR models, it is not only important to make them adjustable and applicable to different application scenarios but also to ensure that they have good performance.

### 5.3. New Loss Functions and Assessment Methods

Currently, most SISR models still rely on the L1 loss or MSE loss as their core dependencies. Although some new loss functions have been introduced, they still struggle to balance image reconstruction accuracy and perceptual quality and cannot achieve ideal results. Therefore, designing more suitable loss functions to resolve this problem remains a key focus of future research. At the same time, existing image quality evaluation methods also have obvious limitations. Some evaluation methods rely more on subjectivity, and some evaluation methods have obvious unfairness in different scenarios. Therefore, it is of great research significance to obtain a more optimal loss function and design an evaluation method that breaks through these limitations.

### 5.4. Mutual Promotion with High-Level Tasks

It is well known that high-level computer vision tasks (such as image classification, image segmentation, and image analysis) are highly sensitive to the quality of input images. Therefore, SISR technology is often used in the preprocessing stage to improve the image quality. Meanwhile, the quality of SR images can significantly affect the performance of these high-level tasks. As a result, integrating image SR with high-level tasks has become a major research topic in recent years. To this end, Zangeneh et al. [197] proposed a novel nonlinear coupled mapping architecture that uses two deep convolutional neural networks to map LR and HR face images into a shared space to achieve LR face recognition. Wang et al. [198] developed a dual SR learning method for semantic segmentation, integrating image SR with semantic segmentation into an end-to-end model. In addition, Xiang et al. [199] significantly enhanced the performance of high-level vision tasks by jointly reducing compression artifacts and performing SR reconstruction. Although these methods have successfully combined SR with high-level tasks and achieved good results, they mainly focus on the output results of high-level vision tasks and rarely consider using feedback from other tasks to further optimize the quality of SR images. Therefore, we suggest using the accuracy of high-level computer vision tasks as a metric to measure the quality of SR images.

### 5.5. Building Real SISR

Although there has been significant progress and improvement in SISR, many algorithm models still fail to achieve the expected results when applied to real-world scenarios. As more and more researchers focus on true SISR, it is expected to become the core direction of this field in the future. The key to achieving true SISR is to build large-scale, high-precision real-image datasets. The application scenarios in the real world are complex and diverse, and various scenarios may be affected by degradation. To construct this realistic dataset, on one hand, a combination of manual and technical methods can be employed, and GANs can be utilized to simulate different degradation patterns and generate finer texture details. On the other hand, blind SISR can be developed for single-image SR reconstruction techniques. Blind SISR can better learn the degradation patterns of images and reconstruct high-quality SR images based on the corresponding degradation patterns. Although blind SISR has achieved good results in image reconstruction, blind SISR methods still need to adapt to different types of scenes and degradation modes. Therefore, the development of blind SISR has important research significance for true SISR. By studying real SISR, the image quality can be improved, image details can be refined, and the image accuracy can be enhanced.

### 5.6. Efficient and Accurate Arbitrary-Scale SISR

In many scenarios, SISR is often used, but most DL-based SISR models have certain limitations, and existing SISR models are often limited in their size. Therefore, developing an SISR model that can adjust to any scale is of great research significance. Although there are currently some SISR methods with arbitrary scales, they often have problems such as complex usage conditions and a lack of flexibility when used. Therefore, this is a valuable research direction that can be further explored in the future.

### 5.7. Considering Features of Different Images

At present, there are already some models in specific fields, but these model methods do not consider different image features and are relatively simple. By utilizing different image features in specific fields, constructing accurate SISR models can effectively improve the performance of these models. Therefore, conducting in-depth research on SISR models using domain-specific images is of great significance. In the future, researching SISR models based on specific domain image features will become one of the focuses of many researchers.

### 5.8. System Security of SISR

In the practical application of SISR technology, system security should be taken into consideration in the future. Kwon et al. [200] proposed an optimal cluster extended intrusion tolerance system, which provides a solution for responding to DoS attacks. The system monitors the response time, CPU usage, and queue length in real time through the cluster controller. Moreover, it adjusts the number of online virtual machines in a timely manner, expands the cluster during the attack, and reduces the cluster after the attack is alleviated, which can reduce resource waste. This mechanism can maintain service quality in both sustained and single-pulse DoS scenarios. SISR systems can draw inspiration from their system security. For example, in monitoring and other SISR-dependent scenarios, similar strategies can be introduced to protect system security and ensure the stability of SR images in the face of attacks.

## 6. Conclusions

STSR is a key research direction in the field of computer vision, with significant research significance and application value. In this review, we first introduced and provided examples of SISR methods based on deep learning. Secondly, the relevant work on SISR was described, and its applications in specific fields were briefly introduced. Afterwards, in order to clearly demonstrate the differences in performance between different models, we listed the reconstruction results of some classic models and compared their performance. Finally, we analyzed the potential issues and future development trends in SISR. We believe that this review can provide some reference value for future research, thereby promoting the development of SISR. 

## Figures and Tables

**Table 1:** Some commonly used datasets.

Name	Usage	Amount	Format
BSDS300 [10]	Train	300	JPG
DIV2K [11]	Train	1000	PNG
Flickr2K [12]	Train	2650	PNG
Set5 [13]	Test	5	PNG
Set14 [14]	Test	14	PNG
Urban100 [15]	Test	100	PNG
Manga109 [16]	Test	109	PNG

**Table 2:** Subjective assessment indicator rating scale.

Quality Score	Degree of Image Distortion	Image Quality
1	The level of distortion is so great that vision is hindered	Very poor
2	Noticeable distortion can be observed in the image	Poor
3	Distortion is observable in the image	Ordinary
4	The degree of distortion is low	Good
5	Visual distortion is not easily noticeable	Excellent

**Table 3:** Comparison of method types for simulating SISR.

Type	Model	Set5 PSNR/SSIM	Set14 PSNR/SSIM	Urban100 PSNR/SSIM	Training Dataset
Efficient Network/ Mechanism Design Methods	SRCNN [7]	30.48/0.8628	27.50/0.7513	24.52/0.7221	T91+ImageNet
	DSRN [37]	31.40/0.8830	28.07/0.7700	25.08/0.7470	T91
Perceptual Quality Methods	MSRN [29]	32.07/0.8903	28.60/0.7751	26.04/0.7896	DIV2K
	SeaNet [111]	32.33/0.8970	28.81/0.7855	26.32/0.7942	DIV2K
	CRN [38]	32.34/0.8971	28.74/0.7855	26.44/0.7967	DIV2K
	EDSR [27]	32.46/0.8968	28.80/0.7876	26.64/0.8033	DIV2K
	RCAN [48]	32.63/0.9002	28.87/0.7889	26.82/0.8087	DIV2K
Additional Information Utilization Methods	SwinIR [69]	32.92/0.9044	29.09/0.7950	27.45/0.8254	DIV2K+Flickr2K
	GRL-B [125]	33.10/0.9094	29.37/0.8058	28.53/0.8504	DIV2K+Flickr2K

**Table 5:** Cost analysis of SISR model calculation.

Model	Platform	Latency	Memory Usage	Application Scenario
DSRN [37]	NVIDIA Jetson Nano	23 ms	0.8 GB	General image SR tasks
CARN [38]	NVIDIA Jetson Nano	15 ms	0.5 GB	Lightweight real-time SR
RDN [194]	NVIDIA Jetson Nano	26 ms	1.2 GB	Medium-precision image SR
MDCN [45]	NVIDIA Jetson Nano	19 ms	0.7 GB	Medium-speed image SR
SRRFN [39]	NVIDIA Jetson Nano	24 ms	1.1 GB	Near-real-time SR
RCAN [48]	NVIDIA Jetson Nano	40 ms	2.5 GB	High-precision image SR
TinySR [195]	Arduino Uno	320 ms	28 KB	Environmental cameras
MobileSR [196]	Arduino Uno	450 ms	31 KB	Low-frequency sensors

## Data Availability

The raw data supporting the conclusions of this article will be made available by the authors on request.
